# Color-specific porosity in double pigmented natural 3d-nanoarchitectures of blue crab shell

**DOI:** 10.1038/s41598-020-60031-4

**Published:** 2020-02-20

**Authors:** Fran Nekvapil, Simona Cintă Pinzaru, Lucian Barbu–Tudoran, Maria Suciu, Branko Glamuzina, Tudor Tamaș, Vasile Chiș

**Affiliations:** 1 Department of Biomolecular Physics, Babeş-Bolyai University, Kogălniceanu 1, 400084 Cluj-Napoca, România; 2 Institute for Research, Development and Innovation in Applied Natural Science, Fântânele 30, 400327 Cluj-Napoca, România; 3 Electron Microscopy Centre, Babeș-Bolyai University, Clinicilor 5-7, 400006 Cluj-Napoca, România; 4 INCDTIM, Donat 67-103, PO 5 Box 700, 400293 Cluj-Napoca, România; 5 Department of Aquaculture, University of Dubrovnik, Ćira Carića 4, 20 000 Dubrovnik, Croatia; 6 Department of Geology, Babeş-Bolyai University, Kogălniceanu 1, 400084 Cluj-Napoca, România

**Keywords:** Biophysical chemistry, Raman spectroscopy

## Abstract

3D-engineered nano-architectures with various functionalities are still difficult to obtain and translate for real-world applications. However, such nanomaterials are naturally abundant and yet wasted, but could trigger huge interest for blue bioeconomy, provided that our understanding of their ultrastructure-function is achieved. To date, the Bouligand pattern in crustaceans shell structure is believed to be unique. Here we demonstrated that in blue crab *Callinectes sapidus*, the 3D-nanoarchitecture is color-specific, while the blue and red-orange pigments interplay in different nano-sized channels and pores. Thinnest pores of about 20  nm are found in blue shell. Additionally, the blue pigment co-existence in specific Bouligand structure is proved for the green crab *Carcinus aestuarii*, although the crab does not appear blue. The pigments interplay, simultaneously detected by Raman spectroscopy in color-specific native cuticles, overturns our understanding in crustaceans coloration and may trigger the selective use of particular colored natural nanoarchitectures for broaden area of applications.

## Introduction

Nikola Tesla would have been surprised to find out that thinking “in terms of energy, frequency, and vibration”, the secrets of the blue crab colors could be elucidated.

Accounted by ecologists among 100 worst invasive alien species in eastern Mediterranean coast^1–5^, seen by seafood producers both as a threat to benthic shellfish cultures^2,4–6^ and a potential new commodity in the invaded areas^4,6^, loved by gourmands as a delicacy, the Atlantic blue crab, *Callinectes sapidus*, poses an increasing interest, as its wasted shells could be potentially turned in valuable by-product, not only because of chitin, proteins^7^ and biogenic calcite content, but also as natural, porous, pigmented biomaterial, yet poorly understood as highly ordered nano-architecture posing inspiration for biomimetics^8^.

Interestingly, *C. sapidus* cuticle simultaneously features bright blue, red and white anatomical exoskeleton counterparts (Supplementary Fig. 1), known to be important cues for mate selection for reproduction^9^. However, considering the large diversity of crustaceans’ coloration, research on blue color origin refers mostly to lobster species *Homarus americanus*^10–13^ and *Homarus gamarus*^14–19^ which is presumed to originate from blue carotenoprotein complexes, where astaxanthin (ATX), an orange carotenoid is non-covalently bonded in crustacyanins^10–21^. UV/Vis absorption band shifted from 480  nm in ATX monomers^12^ to 632, 585–595 and 610  nm in isolated α-, β- and γ-crustacyanin^12^ respectively, suggests the opportunity to exploit vibrational resonance Raman (RR) spectroscopy to selectively detect free or non-covalently bound ATX in custacyanin in intact crab shell by-product.

It is also surprising that the current knowledge on the blue coloration in crustaceans remains controversial, considering the numerous earlier studies^10–12,14,15^, and more recent computational and experimental approaches^13,16–19,22–26^. For instance, Gamiz-Hernandez *et al*.^19^ showed that β-crustacyanin (not α-) is the responsible pigment for the blue color. Based on theoretical calculations^19^, it was found that β-crustacyanin (comprising two stacked ATX molecules), induced a bathochromic shift of ATX arising from the polarization effects and steric constrains of the ATX-protein binding site. On the other hand, based on the available models of crustacyanins, van Wijk *et al*.^17^ proposed a protonation model involving the keto-groups of the ATX terminals near a water molecule. Controversial conclusions underlying the comparative results of the cuticle ultrastructure in lobster *H. americanus* raised additional questions regarding its crystalline nature^27,28^. The “universal” nature of the 3D- hierarchical calcite arrays which follows a twisting plywood path (Bouligand-type pattern) in crustacean exoskeleton has been suggested, based on studies on several species of crabs^8,28–32^, but lacking any connection with the pigments populating the morphological pattern. Katsikini^33^ detected ATX only in *C. sapidus* blue crab cuticle, due to the use of a single laser line (488  nm), which fulfilled the RR conditions for the respective carotenoid only, but suggested that Br and Sr are involved in two different mechanisms contributing to the blue color.

Currently, comprehensive understanding of chemical structure-morphology relationship in blue crab shell is absent, although of high interest for transforming such aquatic porous by-product into added-value material within the blue bioeconomy goal.

Here, relying on combinatory multi-laser RR micro-spectroscopy and imaging, assisted by electron microscopy, X-ray diffraction and computational chemistry, we show that two interplayed pigments and their distribution in the 3D-nanoarchitecture of the plywood path is color-specific and the blue shell of *C. sapidus* is not exclusively determined by the presence blue pigment, astaxanthin bounded in crustacyanin. Moreover the blue pigment is present in other crab cuticle colors, not necessarily blue.

Comparatively, we employed a co-inhabitant, native^2^ species, *Carcinus aestuarii* green crab. The ultimate goal is to properly address such wasted biogenic material within the blue bioeconomy concept.

We hypothesize an additional photonic color component to the overall blue cuticle appearance, sustained by its optical properties^9,33^ (high reflectance between 380–480  nm, selective absorption^33^ between 500–700  nm, weak light transmission of about 9.6% of red *C. sapidus* shell^33^) and, yet limited, RR scattering data of one blue shell^33^. Exoskeleton photonic crystal would be sustained, provided that the ultrastructure periodicity influences the propagation of certain light wavelengths and its dielectric properties are tuned by its morphological biochemistry.

## Results

**Figure 1:**
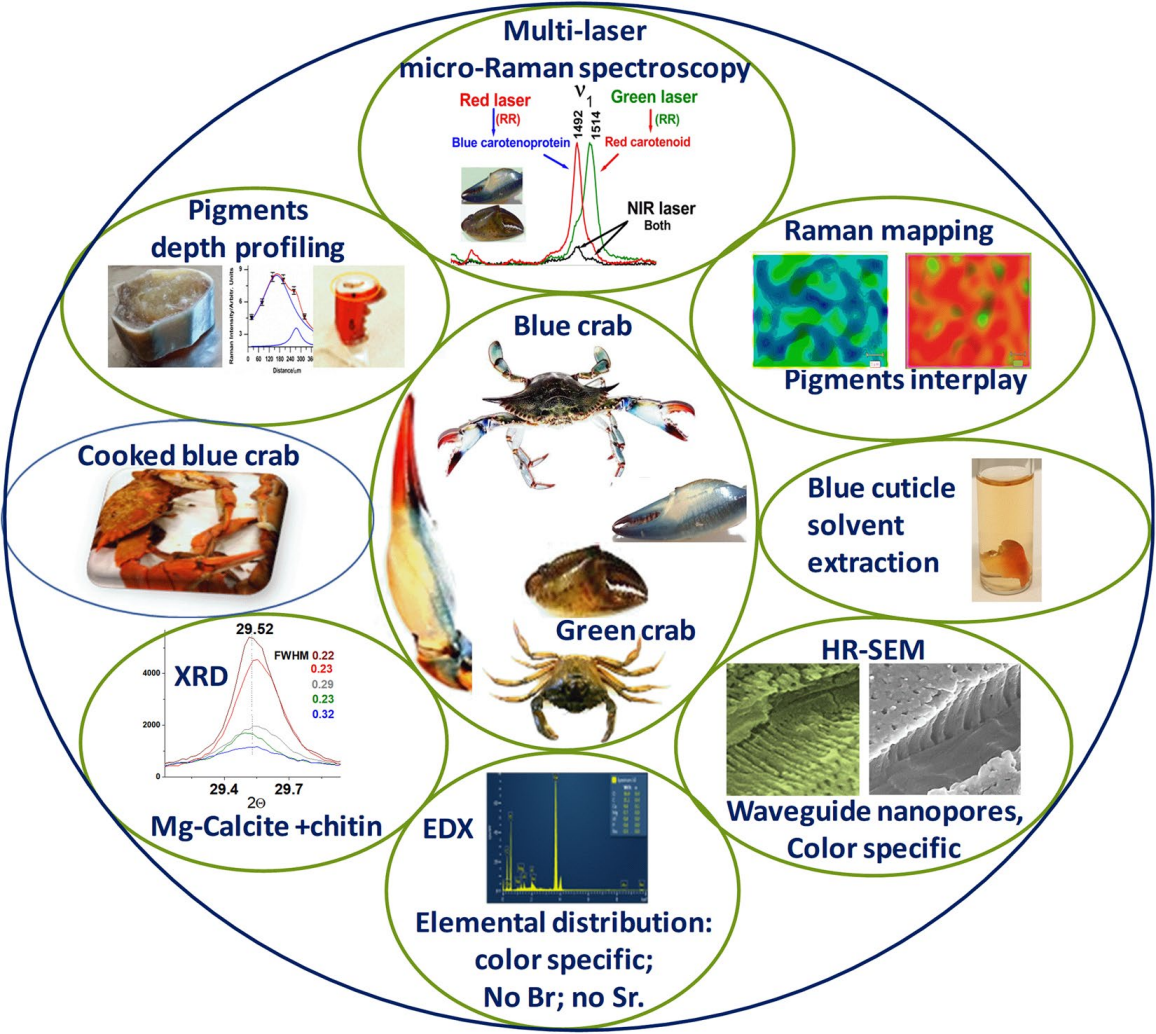
Summary display of the experimental approach comprising resonance and non-resonance Raman micro-spectroscopy, HR-SEM, EDX, XRD of blue, red, white and green exoskeleton of *C. sapidus* and *C. aestuarii* crabs in native form. Blue shells turning red when cooked or solvent extracted is molecularly illustrated by Raman spectroscopy.

The complete experimental approach to extract information on pigments identity and their native distribution in crabs exoskeleton ultrastructure is summarized in the Fig. 1. We combined the unique ability of the multi-laser (resonance) Raman spectroscopic techniques to localize the chemical and structural information, with the morphology from the high resolution scanning electron microscopy (HR-SEM) in conjunction with energy-dispersive X-ray spectroscopy (EDX) and X-ray powder diffraction (XRD) of native blue, red and white shell counterparts of *C. sapidus*, and the green *C. aestuarii* crab. The type, number of samples and experimental purpose of all the samples used in the present study are summarized in the Supplementary Table 1.

### Multi-laser micro-Raman spectra of blue, red, green and white crab shells

Raman spectra collected from each crab shell color are strongly dependent on the laser excitation wavelength and highlights the free or non-covalently bond astaxanthin (ncb-ATX) occurrence in the colored shells when proper laser excitation is applied (Supplementary Figs. 1C, 2). Overtones and linear combinations of pigments Raman modes analysis is given in Supplementary Fig. 3. It was clearly noted that *ν*_1_*(C*=*C)* + *ν*_2_ (C-C) linear combination showed distinct position for ATX and ncb-ATX, the latter being 22  cm^−1^ downshifted. Comparative display of the carotenoid *ν*_1_ *(C*=*C)* Raman band positions in spectra acquired from the four shell types with the three laser lines are showed in the Supplementary Fig. 4.

### The blue cuticle

**Figure 2:**
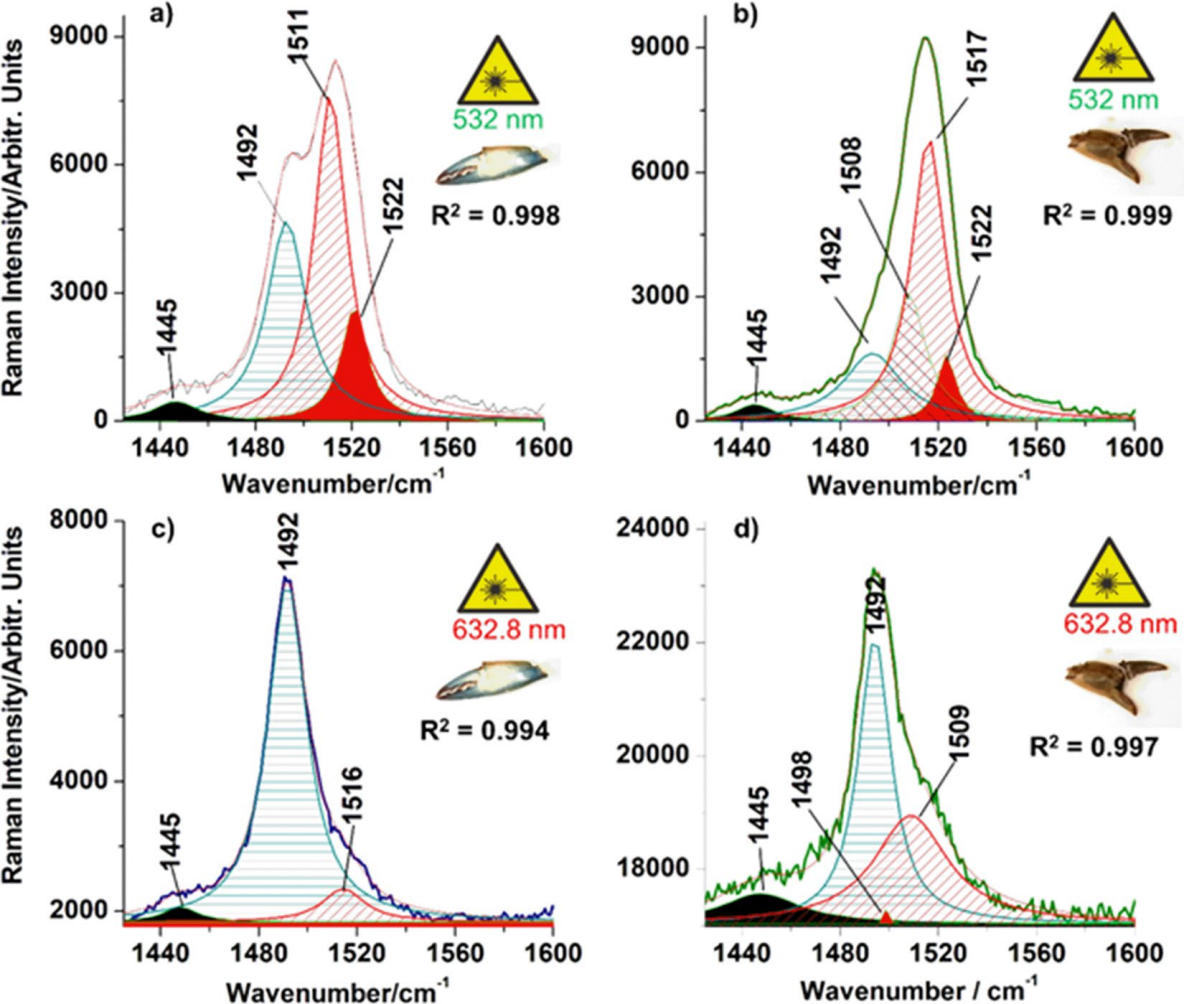
Multi-peak Lorentzian fit of raw RR spectra (1425–1600 cm^−1^ range) acquired from blue *Callinectes sapidus* claw shell (**a,c**), and green *Carcinus aestuarii* claw shell (**b,d**), using two laser lines, as indicated. Profile broadening via concentration was assimilated with pressure broadening, thus, Lorentzian fit was considered for multi-peaks deconvolution. Corresponding coefficients of determination (R^2^) are displayed in the figure. Note the effect of resonant excitation of pigments: deconvolutions of spectra excited with 532  nm featured stronger carotenoid modes above 1500  cm^−1^, while ncb-ATX mode at 1492  cm^−1^ is always dominant as “blue band” in spectra excited with the 632.8  nm line. Additional -CH_2_ mode (filled black band) at 1445  cm^−1^ along with other carotenoid species with minor overall contribution, are highlighted.

The blue exoskeleton fragments of *C. sapidus* cuticles under green laser excitation (532  nm) comprised two Raman band components, one strong band assigned to the ν_1_(C=C) skeletal stretching mode of ATX above 1500  cm^−1^ and a weaker band (or shoulder) in the 1491–1493  cm^−1^ range, attributable to the same ν_1_(C=C) mode in ncb-ATX in carotenoprotein. By contrary, full resonance Raman excitation of carotenoprotein with red laser (632.8  nm) line (which exactly fits crustacyanin extinction maximum^12^ readily revealed the RR mode arising from the ncb-ATX at 1492  cm^−1^ (Fig. 2) and denoted “blue band”. This signature is characteristic to the native carotenoprotein in crab shells and its presence with stronger or weaker intensity describes its relatively different abundance in various colored shells. Notably, its presence in green crab spectrum with weaker intensity (Fig. 2), demonstrates the existence of ncb-ATX in blue carotenoprotein from *C. aestuarii*. In other words, blue pigment is present in green crab shell.

The greater wavelength laser lines (785, 830 and 1064  nm), which are neither resonant to ATX nor its protein complex, always featured a weak shoulder of free ATX band around 1514  cm^−1^ associated with the stronger mode of ncb-ATX at 1491 to 1492  cm^−1^ (Supplementary Fig. 2). All Raman bands recorded from the blue shell with 5 laser lines are listed in Table 1 and their assignments are provided according to our computational chemistry results and literature^11,12,15,17,24,33^.

**Table 1:** Experimental Raman data/cm^−1^ of blue *C. sapidus* claw cuticle obtained with different laser lines (532, 632.8, 785, 830, 1064 nm) comparatively showed with the theoretical calculated Raman modes of ATX and ncb-ATX.

Raman experimental data/cm^−1^ of blue *C. sapidus* cuticle					Calculated Raman modes/cm^−1^: free ATX, ncb-ATX	Vibrational assignment
532 nm	632.8 nm	785 nm	830 nm	1064 nm		
			154	155	—	Calcite (T)
	281	282	284	281	—	Calcite (L)
712	712	712	714	712	—	*v*_4_(CO_3_^2−^) in calcite
—	—	880	—	—	875, 882	ATX *δ*(CH_2_)
—	—	—	—	892	—	α-chitin
953sh	953sh	953sh	—	955	923, 929	ATX *ν*(C-C) + *ρ*(CH_2_) + (CH_3_), α-chitin, *v*(PO_4_^2−^)
963	963	963	963	963sh	972sh, 972sh	ATX *ν*_4_(C-C) + *ρ*(CH_2_) + (CH_3_), α-chitin, *v*(PO_4_^2−^)
1004	1004	1003	1004	1004	992, 997	*v*_3_ ATX, ncb-ATX *ρ*(C-CH_3_), Phe
—	—	—	—	1038	—	proteins (Phe)
1086	1088	1086	1087	1086	—	*v*_1_(CO_3_^2−^) in calcite
1154	1154	1154	1154	1154	1154, 1154	ATX and ncb-ATX *v*_2_(C-C), α-chitin
1190	1190	1197	1190	1190	1186, 1189	ATX, ncb-ATX *ν*(C-C)
	1205	1203	1206	1202	—	α-chitin
	1234	1234	1234	1234	—	proteins (amide III)
1265	1264	1263	1263	1263	1262, 1269	ncb-ATX, ATX *ρ*(C-H), α-chitin
1301	1305	1304	1303		1314, 1307	ncb-ATX in crustacyanin
				1325	—	α-chitin
1385	1375	1374	1375	1375	1384, 1384	ATX, α-chitin
				1412	—	α-chitin
1444	1453	1442	—	1448	1444, 1425	ATX, ncb-ATX *δ*_asym_(CH_2,3_), α-chitin
1492	1492	1492	1492	1492	absent, 1494	*v*_1_(C=C)_out-of-plane_ in
ncb-ATX						
1514	—	1514	1514	1514	1508, absent	ATX, *v*_1_(C=C)_in-plane_
		1517		1517sh	absent, 1522	ncb-ATX *v*_1_(C=C)_in-plane_
—	1603	1604	—	—	absent, 1609	ATX, ncb-ATX *v*(C=C)_out-of-plane_ +
	1621	1619	—	1621	—	Proteins (Trp, Tyr, Phe) + *v*(C=O),), α-chitin
—	—	1657	—	1655	—	α-chitin, proteins (amide I), α-chitin
—	—	‘—	—	1746	—	Calcite (2*v*_2_)
2117	2117	absent	absent	absent	—	*v*_2_ + *v*_4_ in ATX, ncb-ATX
2158	2158	absent	absent	absent	—	*v*_2_ + *v*_3_ in ATX,ncb-ATX
2308	2308	absent	absent	absent	—	2*v*_2_ in ATX, ncb-ATX
absent	2448	absent	absent	absent	—	*v*_1_ + *v*_4_ in ncb-ATX
2518	absent	absent	absent	absent	—	*v*_1_ + *v*_3_ in ATX
absent	2646	absent	absent	absent	—	*v*_1_ + *v*_2_ in ncb-ATX
2668	absent	absent	absent	absent	—	*v*_*1*_ + *v*_2_ in ATX
3028	absent	absent	absent	absent	—	2*v*_1_ in ATX
—	—	2877	—	2878	—	α-chitin *v*(CH_2,3_)
—	—	2937	—	2934	—	α-chitin *v*(CH_2,3_)
—	—	2969	—	2965	—	α-chitin v(CH_2,3_)

Bands assignment is provided according to our computational chemistry results and literature^11,12,15,17,24,33^.

Abbreviations: ATX-astaxanthin, ncb-ATX - non-covalently bound astaxanthin, *v* = stretching, *ρ* = rocking, *δ* = bending, T, L -lattice modes in calcite, sh- shoulder.

The peaks position was reproducible among animals (Supplementary Table 1 and Supplementary Fig. 1C) The relative intensity variability is rather dependent on the local roughness of the shell surface for the incident laser spot and the local interplay of the two pigments ATX and ncb-ATX under focus. Natural, quantitative accumulation of pigments as a function of crab age (all mature crabs) was not aimed here.

We conducted theoretical calculations using the Density Functional Theory (DFT) for the RR spectrum of non-covalently bond ATX (ncb-ATX) and compared it to its free counterpart, to correctly assign the experimentally observed Raman data. Computed Raman modes of isolated ATX featured theoretical *v*_1_ band at 1508  cm^−1^, arising from conjugated in-plane C=C stretching modes, while the calculations for ncb-ATX (ncb-ATX) involved in strong dipole-dipole interactions, which simulated non-covalent binding to the protein carrier, exhibited *v*_1_ mode red shifted to 1494  cm^−1^ (Supplementary Fig. 5). Our calculated *v*_1_ positions are similar to respective experimental modes of α-crustacyanin reported in older and more recent studies^12,15,17^, which indicated good simulation of Raman response of ATX bound in carotenoprotein. The second Raman band themed *v*_2_ assigned to the C-C stretching mode of carotenoid skeletal structure was found at 1154  cm^−1^, being non-sensitive to the non-covalent interaction.

Correlated experimental Raman and theoretical findings clearly suggest that blue shell contains both free and ncb-ATX in carotenoprotein complex. Harmonics and linear combinations of the *v*_1_, *v*_2_, *v*_3_ and *v*_4_ modes of ATX or ncb-ATX in the high wavenumber range (2100–3100  cm^−1^) confirmed the existence of two pigments in the blue shell: they corresponded to ATX modes (Table 1) when the shells were excited with green laser line, and to ncb-ATX, under red laser excitation (Supplementary Fig. 3).

### The red, white or green cuticles

The red and white *C. sapidus* claw cuticles as well as the green ones of *C. aestuarii* have been similarly investigated, as comparatively shown in Supplementary Fig. 2b–d. Excitation with 532  nm line revealed the presence of ATX, while the 632.8  nm line revealed both signal of ncb-ATX and free ATX in red shells. Weak and noisy Raman signal of pigments has been spuriously detected in white shells, too (Supplementary Fig. 2d). Thus, it appears that the presence of ncb-ATX in crustacyanin complex is not limited to blue shells of *C. sapidus* only. In other words, shells containing the complex are not necessarily blue, similar to findings on yellow lobsters^11^, which exhibited strong RR signal of yellow protein, and weak RR signal of ncb-ATX from crustacyanins.

### Three colors, same pigments

Multi-peaks Lorentzian fit of the carotenoid *v*_1_ band in Raman spectra acquired from blue *C. sapidus* and green *C. aestuarii* claw shells (Fig. 2) provides accurate bands composition, revealing other, minor contributions to the main C=C band shape (R^2^ > 0.99). The difference in position of C=C modes above 1500  cm^−1^ can be attributed to stretching modes of conjugated C=C bonds of free carotenoids. The C=C mode arising from the ncb-ATX complex consistently appeared 1492  cm^−1^ (showed as blue shaded band in Fig. 2).

To further clarify the different colors of shells which contain the same pigments, we calculated the relative Raman intensity ratio (R = I_1492_/I_1514_) of the two main modes contributing to the overall *v*_1_ Raman band, assigned to the ncb-ATX and ATX. The near-infrared 785  nm laser line was used for this purpose, as it provides normal Raman scattering only, thus avoiding resonance Raman contribution of either pigment. As the intensity of normal Raman scattering is directly proportional to the analyte concentration, for similar excitation conditions and collecting optics, the intensity ratio (R = I_1492_/I_1514_), gives an estimation of relationship between the content of ncb-ATX and free ATX chromophores in each native shell color. The calculated values of the R ratios defined above are shown in the Table 2 along with their standard errors. Data processing algorithm for the calculation of the R ratio values and the averaged corresponding spectra are shown in the Supplementary Fig. 6.

**Table 2:** Calculated Raman intensity ratios (R) of free- and non-covalently bonded ATX chromophore modes in blue, red and green cuticle, reflecting relative pigment content.

Shell color type	Pigment Raman ratio: I_1492_/I_1514_ (mean ± S.E.)	number of measurements/animal
blue	8.487 ± 0.283	10
red	0.757 ± 0.301	12
green	2.958 ± 0.277	10

S.E.- standard errors.

Note the highest value of R = 8.487 ± 0.283, for the blue color, almost 3 times higher than in the green shell, and more than 11 times higher than in the red shell, meaning that blue shells contain the most ncb-AXT chromophores relative to AXT. Due to the natural inhomogeneity of shell surface color, the considerable standard error values are not surprising. Carotenoid signal was most frequently absent from white shells spectra with non-resonant 785  nm excitation (Supplementary Figs. 2 and 4), however, it was randomly detected in several cuticle fragments under resonance excitation conditions.

**Figure 3:**
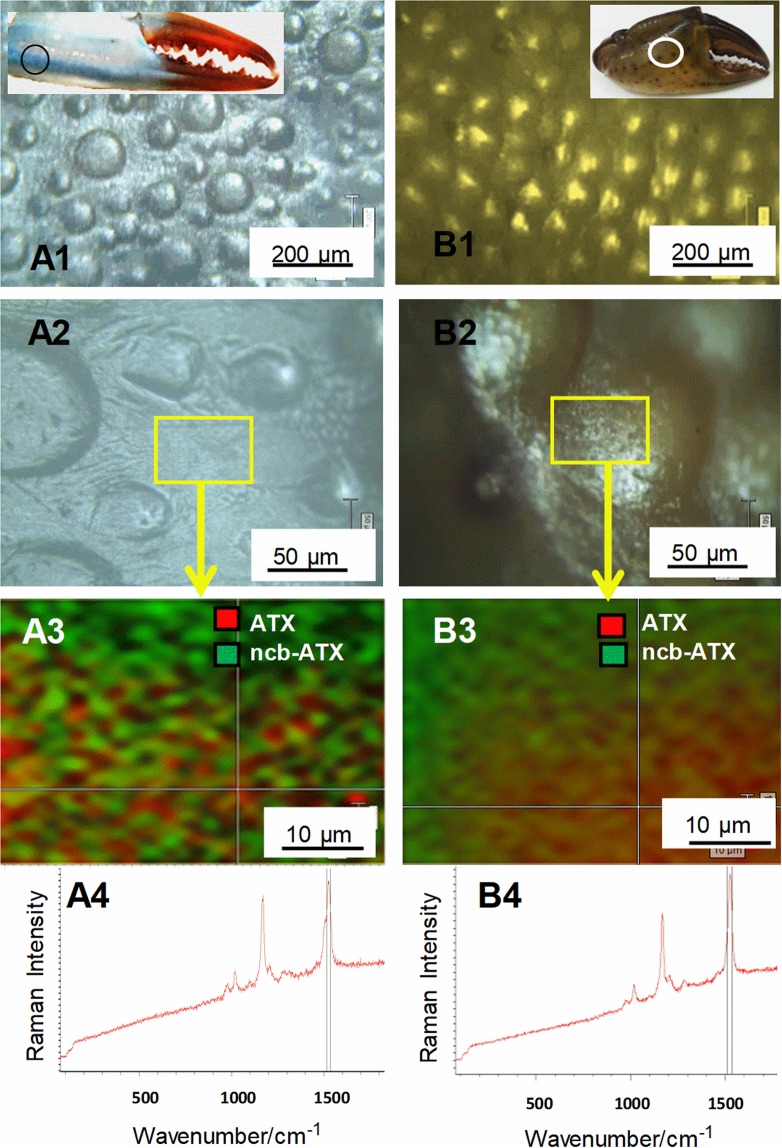
Mapping the Raman intensity distribution of the astaxanthin (ATX) *v*_1_(C=C) band at 1514  cm^−1^ and its non-covalently bound counterpart (ncb-ATX) at 1492  cm^−1^ from spectra collected from the blue *Callinectes sapidus* and green *Carcinus aestuarii* crab cuticle surface. Excitation: 532  nm. A1,2 and B1,2 show the light microscopy images taken via Raman microscope with 5x or 20x objective respectively, while A3 and B3 display the two pigments interplay over the mapped area highlighted in rectangle in A2 and B2. A4 and B4 show the rough Raman signal of the respective cuticle at the cross-hair of the maps. Note the spatial interplay of the two pigments contribution.

Summarizing, different colors of *C. sapidus* and *C. aestuarii* cuticle arise from the balance of co-existent orange and blue pigments. Surface distribution of pigments in crabs cuticle has been further mapped to support the standard errors of R values described above. StreamLine^TM^ Raman imaging approach allowed for rapid generation of Raman maps of surface distribution of ATX and ncb-ATX signal on blue *C. sapidus* and dark green *C. aestuarii* dorsal claw cuticle (Fig. 3). Attempt to do the same analysis for the red shells failed due to the much stronger RR signal superimposed with the background of ATX, which completely covered any trace of the ncb-ATX complex contribution in the StreamLine^TM^ data collection. The obtained Raman images confirmed the co-existence of the both pigments, their inhomogeneity distribution and their interplay.

### Pigments distribution in cuticle cross-sections

**Figure 4:**
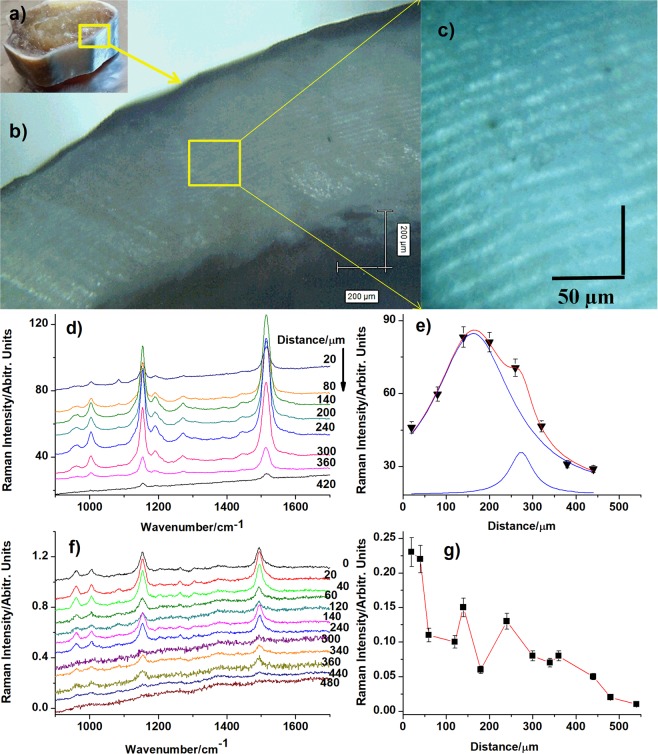
Pigments distribution in the cross section of blue cuticle (**a**) along with the normal-to-surface direction: (**b,c**) micrographs taken via Raman microscope during measurements; (**d**) RR spectra of ATX collected from surface points along the shell transect, from margin to the inner layers (shell depths) from 20 to 420  µm as indicated on each spectrum; (**e**) ATX Raman C=C mode (1514  cm^−1^) intensity distribution against depth; the data fit showed a Lorentzian profile with two components, peaking at 130 and 260 μm respectively; (**f**) RR spectra of ncb-ATX taken from cross section surface (shell depth from 20 to 240 μm); and its main band (1492  cm^−1^) intensity variation along cross section (**g**), showing the highest intensity in epicuticle. Error bars show the standard deviation.

Biogenic material fractures were obtained by cleaving shells of specific color. Thus, the fracture surface analysis by Raman micro-spectroscopy provided information on the pigments presence in shell cross section. Typical raw RR spectra (532  nm excitation) collected from blue shell cross section at 20, 80, 140, 200, 240, 300, 360 and 420 μm distance from margin showed in the Fig. 4d), revealed characteristic signal of ATX superimposed with strong background. Red laser excitation (632.8  nm) of the same cross section points provided characteristic RR signal of ncb-ATX with even stronger background, (Fig. 4f). However, several typical RR spectra from 0 (surface), 20, 40, 140 and 240 μm distance from shell margin still could be obtained under similar acquisition conditions (1  s, 1 acquisition, 10  mW, 100 x objective).

The ATX and ncb-ATX exhibited well-defined Raman signal collected from the fracture surface and the intensity evolution of carotenoid *v*_1_ band was tracked, as showed in the Fig. 4.

The fit of collected data points revealed two distinct depths with high ATX content in blue shell, the first maximum being around 130 μm, and the second at the depth of about 260 μm (Fig. 4). Red and green cuticles exhibit different depth profile of ATX band intensity (Supplementary Fig. 7). Carotenoid content, proportional with the intensity of the *v*_1_ Raman band (Supplementary Fig. 7c,f), appeared with variable distribution, with higher content at 10 to 20 μm beneath cuticle surface in both red and green shells, followed by another peak at about 50 μm depth in red and 100 μm in green shell. The data suggest lower, but consistent carotenoid presence towards endocuticle inner layer. Cross section of blue cuticle tracked with the 632.8  nm line revealed highest intensity of ncb-ATX in epicuticle, following a rapid decrease within 20 μm depth, than further slow decrease between 20–140  µm and very weak intensity up to 240 um depth (Fig. 4). Previous immunostaining approach to determine pigments distribution in spiny lobster *Palinurus cygnus*^34^ located crustacyanins only at the depth of 7 to 8 μm beneath cuticle surface and in hypodermis of the cuticle.

### Blue and green shells turn red-orange when immersed in boiling water or after ethanol extraction, leading to the disappearance of the ncb-ATX resonance Raman band

Additionally, when blue, red and green claw shells were immersed in boiling water (100 °C) or ethanol to probe pigments extraction, Raman analysis of same shell fragments showed that intact ncb-ATX in crustacyanin complex is prerequisite for maintenance of shell native color. Each treatment resulted in complete dissociation of the complex (Supplementary Fig. 8), leaving behind abundant free AXT, which gives intense orange color aspect to the shells. In addition, ethanol extraction appeared simple method of obtaining valuable carotenoid-enriched solution from the shells, which may further be used for carotenoid purification, while preserving the intact porous calcite morphology. (i) *C. sapidus* blue and red, and *C. aestuarii* green claw shells changed their native color into red-orange after few seconds of immersion into boiling water (Supplementary Fig. 8a,b). White couterparts of *C. sapidus* claw remained unchanged. Only ATX could be detected in the treated shells (Supplementary Fig. 8b), while the ncb-ATX RR band was absent. It results that crustacyanin complexes released ATX at boiling water temperature, followed by diminishing of darker color shade, while free AXT, which would not be affected by short exposure to high temperature, remained unaltered. (ii) Likewise, the blue *C. sapidus* native claw shell turned pinkish red after 14 days of carotenoid extraction in ethanol. The marker RR band of ncb-ATX was not detectable in shells after extraction, indicating total dissociation of the ATX-crustacyanin complex by ethanol. Furthermore, the additional RR analysis of extract solution showed that only free ATX may be retrieved from crab shells after exposure to ethanol, due to the dissociation from crustacyanin under the effect of polar protic solvent treatment.

### Mineral composition of the crab claws

Fourier-transform Raman (FT-Raman) data obtained with the 1064  nm laser line excitation revealed the prominent symmetric stretching mode of carbonate ν_1_(CO_3_^2−^)^35^ in calcite, ranging from 1085 to 1088  cm^−1^, the ν_4_ *in plane* bending mode^36^ around 712  cm^−1^ and well-defined calcite lattice bands at 155 and 281  cm^−1^ in all cuticle color types (Supplementary Fig. 9). The intensity and full width at half maximum (FWHM) values of the^-^ *v*_1_(CO_3_^2−^) stretching mode indicated the highest calcite crystallinity in red dactyls, followed by green dactyls, white, and blue palm shells. Significant broadening of the *v*_1_ mode to the lower wavenumbers, paired with broadening of the *v*_4_ and lattice modes indicates the co-existence of amorphous^37^ CaCO_3_ in blue and white shells, while broadening of *v*_1_ to higher wavenumbers indicates small inclusions of Mg atoms in calcite crystalline lattice^36^. The area ratio r of the two Raman band components of calcite stretching mode in blue shell showed a value r = area_(1085)_/area_(1075)_ of 2.82 (Supplementary Fig. 9). Additionally, α-chitin^38,39^ could be identified according to its characteristic Raman fingerprint bands at 892, 955, 1059, 1111, 1147, 1202, 1263, 1325, 1375,1412, 1448, 1621, 1655 cm^−1^ as well as the CH_2,3_ stretching modes at 2878, 2934 and 2965  cm^−1^ (Supplementary Fig. 9, Table 1). Weak protein bands^35^ were observed at 1001, 1038, 1234  cm^−1^, and possibly contributing to bands at 1621 and 1655 cm^−1^ (Table 1). Carbonates, α-chitin, proteins and carotenoids all account for the observed FT-Raman bands with variable relative intensity from one beamed point to another. Such relative distribution over the cuticle surface suggested by FT-Raman analysis is confirmed by the Raman maps generated from the signal-to-baseline approach in fast StreamLine^TM^ imaging of blue cuticle surface (Supplementary Fig. 10) excited with the NIR line at 785  nm.

**Figure 5:**
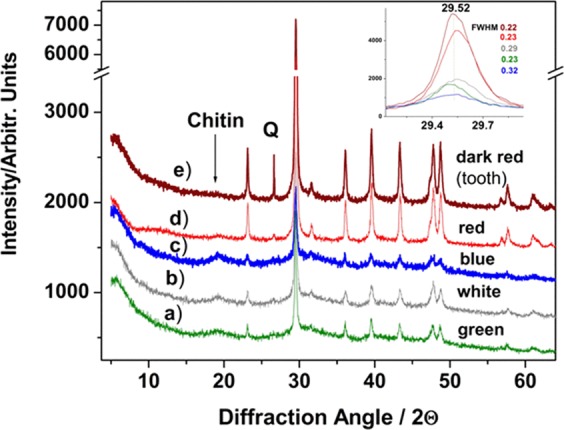
XRD diffractograms of the powdered crab cuticles as indicated, showing the Mg-calcite pattern. FWHM values of the main peak are inserted. Additional signal from crab tooth (e) is showed to highlight its higher crystallinity compared to the claw cuticles. Q- denotes quartz peak probably originated from benthic diatoms inhabiting the cuticle and/or spurious sand micro-grains (especially in the claw teeth). The weak band centred at 19° 2θ was assigned to α-chitin.

The X-ray powder diffraction data (Fig. 5) confirm the presence of low Mg-calcite through the (1 0 4) peak at d = 3.02, compared with 3.035 in pure calcite, according to results of Boßelmann *et al*.^40^ reported for *H. americanus* and *Cancer pagurus*. The highest Mg-calcite pattern was observed in red claw (even higher for its corresponding teeth) followed by the white, green and blue cuticle, as showed in the Fig. 5. The FWHM (showed in the insert of Fig. 5) varied from 0.23° in red and green, 0.29° in white to 0.32° 2θ in the blue cuticle. The dominant Mg-calcite of variable crystallinity is associated with small amounts of quartz (probably originated from benthic diatoms inhabiting the cuticle and/or spurious sand micro-grains) in some of the shell parts (especially in the red shell and claw teeth) observed at 26.67° 2θ, while a weak, broaden band centred at 19° 2θ was assigned to α-chitin^41,42^.

### Different crab color cuticles morphology showed systematic differences at ultrastructure level

**Figure 6:**
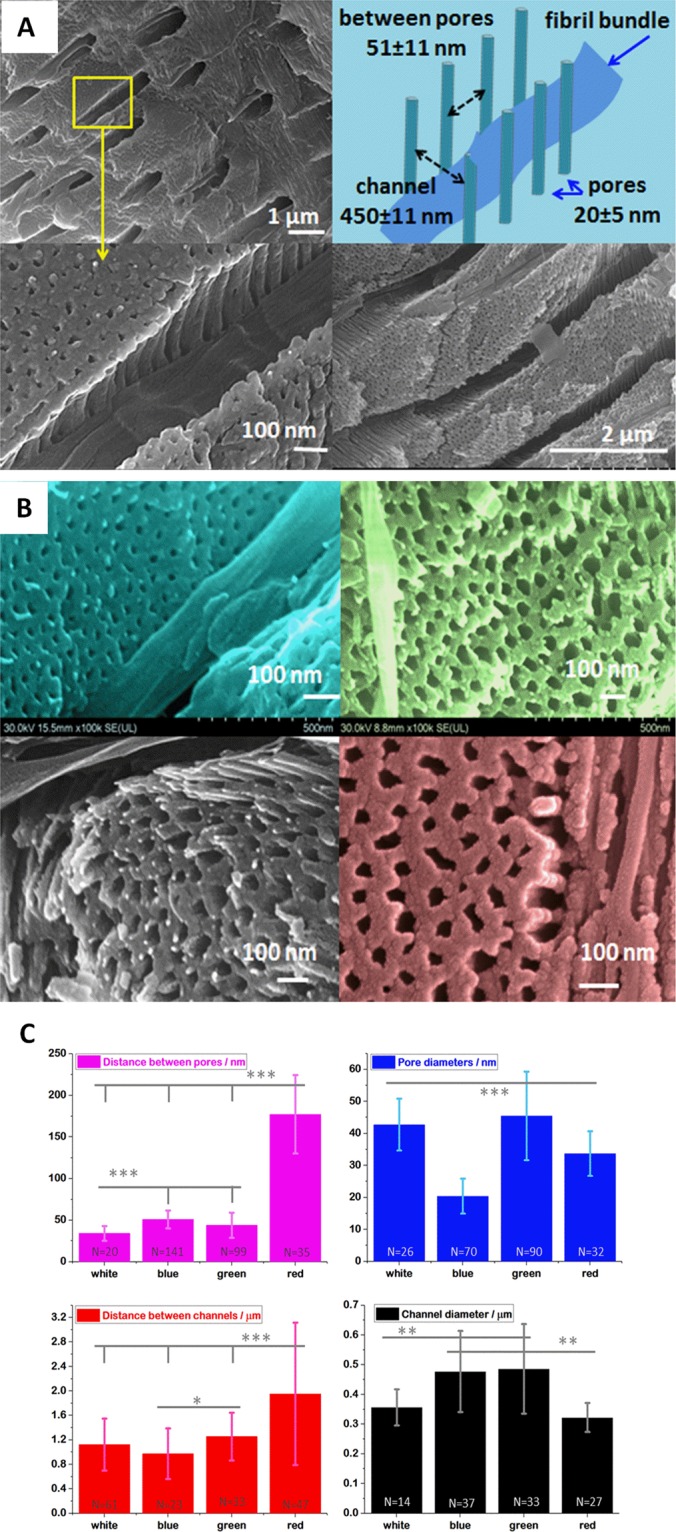
(**A**) Representative SEM images collected from blue cuticle, highlighting the channels and the regular arrays of nano-pillars separated by pores in the ultrastructured channel walls. Schematic representation of the pores and channels arrangement is displayed in the top right corner. The channel width of about 450  nm is comparable with the blue light wavelength. (**B**) Comparative SEM images from the four coloured shell, blue, green, white and red, showing nanoscale details of color-characteristic pores. (**C**) The averaged distances between pores and canals and their diameter for the white, blue, green and red cuticle, as indicated on each graph. Error bars show the standard deviation; statistical difference for values of p ≤ 0.05 were considered significant (and represented by*), p ≤ 0.01 very significant (and represented by**), p ≤ 0.001 extremely significant (and represented by***).

Performing morphology analysis, an ordered, stacked mineral structure was observed, similar to those reported for other crab species^8,28–32^. A series of SEM images collected from fractured cuticles of blue shell is showed in the Fig. 6A. Comparative morphological details of the white and red cuticle of *C. sapidus* and green cuticle of *C. aestuarii* are given in the Fig. 6B. The averaged distances between pores and canals and their diameter for the white, blue, green and red cuticle are distinct, as showed in the Fig. 6C.

The mineral layers are stacked quasi-parallel to the outer surface and their arrangement defines the epicuticles (detail of detached epicuticle morphology is showed in the Supplementary Fig. 11 for the green shell), the exocuticle, where the layers are thinner, and the endocuticle, where much thicker layers are observed. The mineral layers 3D nanoarchitecture is crossed by pore canals hosting the chitin-protein fibril bundles which follow a helical path normal to the cuticle surface. The canals determine the “macro-pores” which cross the successive mineral layers (Supplementary Fig. 11).

Such morphology explains the strong Raman scattering signal of pigments collected in back scattering configuration normal to cuticle surface, or weak signal when the collection direction is normal to the fracture plan (or parallel with the cuticle surface). Furthermore, the spotted pattern of the pigments interplay from Fig. 3 might be consistent with the canals distribution, each canal being populated with chitin-proteins fibrils, thus strong Raman signal in spotted organic areas has been observed along radial fibrils direction (from cuticle top) surrounded by mineral signal. In general, the signal was constantly weaker when cross section was Raman investigated (as showed in the Fig. 4 and Supplementary Fig. 7). The randomly oriented microcrystals and micro-canals of the shell at the fracture surface exposed for Raman acquisition may have caused increased background, merely impossible uniform focus of adjacent points, resulting band broadening, drastically increased background and decrease of Raman signal-to-background ratio.

The pore canals exhibit complex arrangements, showing grating-like pattern of parallel mineral nanopillars (Fig. 6A), which also follows the helicoid path orientation. This structure, which has nanometer-scale arrangements at subwavelength optics, might be essential for selective light waveguiding through the canals, making the blue shell absorbance peaking between 550-650  nm, red between 400–500  nm, while the white one above 600  nm, which corresponds with the diffuse reflectance data previously reported^9,33^. To further check any optical correlation, we measured a series of selected cuticle morphological features (Table 3). Each cuticle color revealed different width of pore canals and different distances between rows of pore canals, which in turn results in different grating constants responsible for manipulation of light. A schematic representation of these canals and pores acting as 3D-gratings is showed in the Supplementary Fig. 11.

**Table 3:** Comparative size of the measured crabs cuticle morphology elements. The data are listed as average values ± standard deviation; n - numbers used for variance analyses using ANOVA combined with comparison test.

Morphology element/unit	White cuticle	Blue cuticle	Green cuticle	Red cuticle
Canal diameter/µm	0.356 ± 0.061 n = 14	0.477 ± 0.136 n = 37	0.486 ± 0.151 n = 33	0.321 ± 0.0489 n = 27
Distance between canals/µm	1.122 ± 0.423 n = 61	0.973 ± 0.414 n = 23	1.253 ± 0.391 n = 33	1.951 ± 1.163 n = 47
Pore diameter/nm	42.739 ± 8.110 n = 26	20.406 ± 5.448 n = 70	45.475 ± 13.834 n = 90	33.695 ± 6.978 n = 32
Distance between pores/nm	34.024 ± 8.891 n = 20	50.804 ± 10.780 n = 141	43.909 ± 15.404 n = 99	177 ± 47 n = 35
Epicuticle thickness/µm	2.285 ± 0.453 n = 18	1.189 ± 0.328 n = 18	3.471 ± 1.299 n = 30	3.268 ± 1.384191 n = 18
Exocuticle thickness/µm	30.727 ± 5.659 n = 21	29.174 ± 2.759 n = 6	47.030 ± 12.317 n = 22	25.050 ± 12.158674 n = 15
Endocuticle thickness/µm	646.234 ± 135.972 n = 11	510.447 ± 11.905 n = 6	200.615 ± 8.099 n = 4	1033.23 ± 221.34 n = 34

Thus, the blue cuticle showed canals diameter (averaged values) of 477, green 486, red 321 and white 356  nm while the distance between canals was 977  nm in blue, the lowest among the other color cuticles exceeding 1 μm (Table 3, Fig. 6C). The wall structure of canals showed the thinnest pores diameter in blue cuticle of about 20.4  nm, and the densest pore 3D-array. Combined with the thinnest epicuticle (Supplementary Figs. 12 and 13), the blue shell appeared morphologically distinct.

The finding overturns our understanding of the 3D-nanomaterial with Bouligand patterns from blue crab shells, simultaneously exhibiting blue, red and white color.

Semi-quantitative elemental composition revealed by scanning electron microscopy and energy dispersive X-ray spectroscopy (SEM-EDX) supports the shell color distinct feature. Relative mass fractions (wt%) for the 5 main elements (Ca, C, O, Mg and P) were obtained from 2 to 4 scans (data plotted from individual column colors, Supplementary Fig. 13) corresponding to epicuticle, exocuticle and endocuticle of *C. sapidus* blue, red and white and *C. aestuarii* green cuticle, respectively. Semi-quantitative EDX elemental analysis (Supplementary Table S2) confirmed the FT-Raman and XRD conclusions of low Mg-calcite, by consistently revealing Mg in all shell color types and cuticle layers alongside Ca, C and O as main elements, trace of P, K, Na, and spurious S and Cl. Furthermore, detection of P in all shells, correlated with the Raman band at 963  cm^−1^ containing contribution of PO_4_^2−^ mode (Table 1), could indicate the presence of trace amount of carbonated hydroxyapatite, which is known to occur in crustacean cuticles^43,44^. However, this supposition is not supported by the XRD data showing that any phosphate mineral was under detection limit in the calcite matrix. Overall, the blue cuticle showed completely distinct composition, revealing the lowest Ca level in epicuticle, further increasing in exocuticle and showing an unusually high amount (up to 71.6  wt%) in endocuticle; lowest C, O and Mg level in endocuticle and highest P content in exocuticle. Such feature, could suggest distinct protective role of endocuticle as the thickest load-bearing layer. In line scan of elemental composition, EDX data constantly showed higher content of C associated to corresponding lower content of Ca, Mg and P, particularly in epicuticle, which mostly contains amorphous minerals and waxy lipoproteins^45^. None of the collected EDX spectra showed any trace of Br or Sr to support the previous consideration^33^ on the role of these elements in blue crab coloration (Supplementary Table S2).

### Statistical analysis

We conducted t-test and one way ANOVA by comparing two groups at a time.

We first conducted one way ANOVA to four groups (white, blue, green, red) and within certain groups (comparing for certain characters) there were no significant differences. Then we compared series of two groups by ANOVA and by t-test as well, to differentiate the significance between groups. The results were the same (p values) using t test or using ANOVA. Multiple t test was not used here.

Statistical analysis revealed the following: channel diameter measurements gave highly significant difference when comparing white-blue (p = 0.0025), white-green (p = 0.0033), blue-red (p = 0.0001) and green-red (p = 0.0001) but no significant difference between white-red (p = 0.0586) and blue-green (p = 0.8017). The distance between channels have extremely significant difference between white-red (p = 0.0001), highly significant between blue-green (p = 0.0127), blue-red (p = 0.002), green-red (p = 0.0014) but no significant difference comparing white-blue (p = 0.15), white-green (0.14). When we analyzed pore diameters we obtained extremely significant difference comparing white-blue (p = 0.0001), white-red (p = 0.0001), blue-green (p = 0.0001), red-green (p = 0.000), but no significant difference between white and green (p = 0.3392). The distance between pores have extremely significant difference between all groups: white-blue (p = 0.0001), white-green (p = 0.0065), white-red (p = 0.0001), blue-green (p = 0.0001), blue-red (0.0001), green-red (p = 0.0001).

Comparing exocuticle thickness between pairs, all groups revealed extremely significant difference: white-blue (p = 0.0001), white-green (p = 0.0003), white-red (p = 0.0045), blue-green (p = 0.0003), blue-red (p = 0.002), except when we compared green and red (p = 0.651) exocuticles where there was no significant difference. The epicuticles gave statistically relevant differences when comparing green with white (p = 0.0001), blue (p = 0.0001) and red (p = 0.0001), but for the others: white-blue (p = 0.3028), white-red (p = 0.0814) and blue-red (p = 0.1696), no relevant difference was observed. Endocuticle thickness had extremely significant difference between all groups (p = 0.0001), and good statistical relevance for the white-blue comparison (p = 0.0295).

### Discussions and outlook

Red ATX and its blue protein complex presence not only in blue, but in red or green crab cuticle is a proof of the complex mechanisms underlying the crustacean colors. Since the previous studies targeted mostly lobsters (*H. americanus* and *H*. gamarus)^10–19^, little is known about the occurrence and localization of astaxanthin-crustacyanin complexes in various crustacean species. Nevertheless, a genetic study conducted to prospect occurrence of genes that encode crustacyanin subunits^34^ established that crustacyanin is a lineage-specific adaptation developed by common ancestors of certain crustacean groups, however, the genome of *C. sapidus* was not considered.

Considering the chemical composition described via Raman, XRD and SEM-EDX, the pigments identity, their interplay and distribution in blue crab cuticle is clearly related to its biomineralization, which is different in blue, red white or green cuticles. Although the variability is high, as reflected by the standard deviation of data, the dimensions of the morphological micro- and nano canals and pores organized in regular 3D-nanoarchitecture clearly influence the selective light absorption, transmission, reflection, scattering or diffraction phenomena, thus, contributing to the final color appearance. The chemical composition and morphology resulted from non-resonance micro-Raman analysis is supported by the XRD and SEM-EDX results. It appeared that the crab cuticle color is not governed by the pigments chemistry only, but also by their distribution in specific 3D nano-architecture, which can be considered as a tri-dimensional supergrating.

Combined Raman spectroscopy approach including the proof of pigment identity by analysing Raman overtones and linear combinations, although never applied in earlier Raman studies^12,15,17^ on carotenoids or carotenoproteins, seemed effective for native exoskeleton pigments analysis. For larger scale approach, Raman tools seemed effective for wasted crustacean materials assessment for their re-use following the blue bioeconomy purpose.

Although the cuticles exhibit low transparency^9,33^ for visible range, certain light wavelengths can reach, propagating through the channels and pores or scattering/diffracting on their typical 3D-nanoarchitecture. Thus, the blue shell showed a series of individual ultrastructural characteristics of pores, canals and nanopillars network which differentiate from the other shell colors. The aragonite polymorph reported in *C. sapidus* exoskeleton harvested on Aegean coast^33^, as well as the presence of Sr and Br to influence the crystalline structure and color^33^ is not sustained by the present data (Supplementary Table 2) on blue crab harvested on Adriatic Sea coast. Our Raman &XRD data unambiguously showed calcite polymorph only.

Although the complex optical effects are difficult to be simultaneously assessed taking into account the pigments localization and weak light transmission in conjunction with the refraction, scattering, absorption, or diffraction and interference phenomena in the 3D-super-array, their co-existence is supported by the physical-chemical and morphological characteristics. For example, chitin, a transparent polymer for the visible wavelength can modify the weak light transmission via refraction and scattering along the canals hosting the fibril bundles.

The highly ordered porous calcite 3D-supergrating, specific to each crab cuticle and gradually populated with balanced pigments appeared more complex than the extensively investigated structural colors in insects. In the latter, the optical properties of their cuticles stem from highly ordered Bouligand pattern comprising line gratings and/or vanes of their wings structure, with helicoid stacking of chitinous fiber layers^46,47^. The circular polarized light reflection which has been observed by Michelson in certain insects exoskeleton a century ago and supposed to be due to a “screw” structure (later named Bouligand pattern) could add additional contributions to the overall properties^43,44,48^ and color appearance not only for human vision, but also for crustacean’s vision. Other proteins can contribute with their specific absorption and/or fluorescence emission along the canals confined between cladding nanopillars walls network, while the nanopillars themselves defining the canals walls structure can act as 3D diffraction grating whose operability in certain diffraction order still has to be understood.

*C. sapidus* cuticle may inspire further biomimetics avenues, since there are no clear-cut borders between blue, red and white shells. Since the crab cuticle can manipulate light with its combined chemical structure and morphology, light absorption and propagation in wavelength dependent manner could warrants attractive applications of the porous magnesian calcite supergratings. In the light of present findings, crab shells could potentially trigger the starting point of new, effective, advanced materials that could serve as porous layer for preventing infections spreading^49^, control molecular solutions loading and slow releasing, bacterial therapies, or develop new, effective, porous biostimulants for soils remediation and agricultural strategies, in line with the most innovative blue bioeconomy approach^50^.

## Conclusions

Non-destructive multi-laser micro-Raman spectroscopy and imaging revealed that native claw shells in the Atlantic blue crab *C. sapidus* from invaded Adriatic Sea contain both the aggregated and the non-covalently bound astaxanthin in carotenoproteins. Further investigation of red and white *C. sapidus* and green *C. aestuarii* claw shells revealed that the two pigments are ubiquitously present in investigated shells, even in seemingly unpigmented white shells, albeit to much lesser extent. Thus, non-covalently bound astaxanthin in crustacyanins is not the only factor responsible for dark blueish and purplish color, but rather participates in formation of apparent color alongside free carotenoids and the 3D nanostructured regular array of Mg-calcite on organic scaffold. The pigments were found to co-localize over the cuticle surface and in its cross-section, with highest distribution in exocuticle layer. *C. sapidus* and *C. aestuarii* also may have a structural color component, derived from the 3D-nanostructured regular array of Mg-calcite on organic scaffold.

## Materials and Methods

### Preparation of crab shells

Atlantic blue crabs (*C. sapidus*) and Mediterranean green crabs (*C. aestuarii*) were obtained from fishermen who caught them by gillnets and traps in Parila Lagoon (southeast Adriatic Sea, Croatia)^2,6^. Crabs were brought to laboratory and frozen at −20 °C until needed. Before analysis, the crabs were thawed and their claws were dissected and cleaned from soft tissue that was inside using scissors, tweezers and tap water. Shells were then thoroughly washed in cold running tap water. 10 mature *C. sapidus* individuals (carapace width about 12  cm), comprising 5 males and 5 females (Supplementary Table S1), differing in color pattern and shades (Fig. 1) were selected (males feature only blue and white shells, while females blue, white and red shells). Additionally, 3 mature *C. aestuarii* individuals were similarly considered. Shells were further cut into smaller pieces to fit the size requirements for respective analysis technique. Sample pieces of cuticle were consistently taken from mature specimens featuring the most intense coloration of the claw palm. Red and green samples were taken from claw dactyls, as illustrated in several samples depicted in the Supplementary Fig. 1. The type and number of samples used in the present study is summarized in the Supplementary Table 1.

### Theoretical calculation

Geometry optimizations and Density Functional Theory (DFT) frequency calculations of the isolated astaxanthin molecule and astaxanthin near water molecules were performed with the Gaussian 09 software package^51^, at B3LYP/6-311 G(d,p) level of theory^51–56^. The solvent effects have been considered by using the implicit SMD model^56^.

### Multi-laser micro-raman spectroscopy analysis

Micro-Raman analysis was conducted on a Renishaw InVia confocal Raman microscope (Renishaw, UK). Four laser lines were used on this device: 532  nm excitation was provided by a Cobolt diode pumped solid state (DPSS), air cooled laser, He-Ne- laser provided the 632.8  nm excitation, and near-infrared excitation was provided by two diode lasers emitting at 785 and 830  nm. Spectral resolution was 0.5  cm^−1^ for visible laser line and 1  cm^−1^ for NIR lines. WiRE^TM^ 3.4 software (Renishaw, United Kingdom) was used for data acquisition. The spectra were acquired either in single-point scan, or in StreamLine imaging mode (described later), on a mobile XYZ HSES (high-speed encoded) stage.

Bruker Equinox 55 FT-IR spectrometer with integrated FRA 106/S IR Raman module was used for to record FT-Raman spectra. A Nd:YAG laser emitting at 1064  nm with 350  mW laser power was used for excitation, and signal was detected by a liquid nitrogen cooled Ge detector. Spectral resolution was 4  cm^−1^ and 300 scans were co-added.

Due to specific sample surface characteristics (as discussed in Results section) various signal intensity was recorded from different parts of shells. Nevertheless, to keep signal acquisition parameters uniform, we used single scans with integration time and laser power to 1 s and 100  mW, respectively, for short-range (100–1800  cm^−1^) and 10  s and 20  mW for extended range scans (100–3200  cm^−1^), for 532, 632.8, 785 and 830  nm lines. The inhomogeneity of claw surface, both in relief and pigment content, made it challenging to record optimal signal-to-noise ratio. Spectra acquired with 532 and 632.8  nm excitation lines featured incremental fluorescence background typical for carotenoid pigments while spectra acquired with 785, 830 and 1064  nm laser line excitation featured low or negligible spectral background.

Spectra of liquid sample, i.e. the astaxanthin-enriched ethanol extract, were acquired on a DeltaNu compact, dispersive Raman spectrometer (Intevac, United States). The 532  nm excitation line was used, and spectra were recorded with 8  cm^−1^ spectral resolution from samples prepared in 1  ml vials, using the NuSpec software (Intevac, United States).

To support the presented data, Raman spectra were collected from 10 blue crabs (5 males and 5 females) and 3 green crabs. Examples of shell fragments are showed in Supplementary Fig. 1 and the description of measurements are summarized in the Supplementary Table 1. Each blue crab individual resulted in dozens of shell fragments of blue and white (males) and blue, red and white (females). The shell fragments were sub-fragmented for different analyses, as described.

### Streamline raman imaging

Raman images were produced on surfaces of blue and red *C. sapidus* and green *C. aestuarii* claw shells using the StreamLine^TM^ image acquisition facility of the Renishaw instrument. Both the 532 and 785  nm excitation lines were used separately, paired with 20x objective (NA 0.35). Firstly, a rectangular area to be analysed, 90 × 90  µm in size, was defined on the live video image. This area resulted in 9000 spectral acquisition points. The Raman image was acquired under a continuous laser beam by automatic movement of the stage to achieve 1 s exposure time per image pixel. The *v*_1_ mode of free ATX, centred around 1514  cm^−1^ and the *v*_1_ mode of ncb-ATX, centred around 1492  cm^−1^ were selected for creating separate images under the “signal to baseline” criterion. The images were then overlaid to produce composite pigment distribution maps.

### Spectral data processing

Raman and XRD spectra were processed in Origin 6.1 software (OriginLab, United States). Background subtraction was applied when needed, by creating a user-defined, automatic, 10 points baseline function and iteratively modifying it using the subtraction facility of the Origin software. Lorentzian fit multi-peak was applied for the Raman spectra in the 1425 to 1600 cm^−1^ range, estimating the initial half width and obtaining the peaks components position, area and center and yielding the coefficient of determination R^2^ higher than 0.99. Calculation of Raman intensity ratio (R) of ncb-ATX band to ATX was done after background subtraction from spectra acquired from different shell color types with 785  nm excitation and subsequently comparing the intensity of the mode at 1492  cm^−1^ (ncb-ATX) and the mode at 1514  cm^−1^ (ATX) as R = I_1492_/I_1514_ (Supplementary Fig. 6). Normalization of spectra was done by dividing all y data of spectral datasets by the intensity value of the carbonate stretching band.

### Additional shells treatment

#### High temperature denaturation of astaxanthin-crustacyanin complex

Blue, red and white pieces of *C. sapidus* and green pieces of *C. aestuarii* claw cuticle were placed into Petri plate containing boiling water. Water was heated on a magnetic hot plate magnetic stirrer to 100 °C, and pieces of shells were kept in boiling water until their color changed to orange. Shells were then taken out of water using tweezers, dried and subsequently analyzed with the Raman microscope. Raman spectra were acquired from treated shells using the 532, 632.8, 785 and 1064  nm laser lines, and spectra were compared to those acquired from native blue and green shells.

#### Crude ethanol extraction of shell pigments

Pieces of blue shells from *C. sapidus* claw were placed in vials containing 4  ml of ethanol. Raman analysis of both shells and the supernatant was conducted 14 days after the beginning of extraction. Shells and carotenoid microcrystals were analyzed with the Renishaw Raman microscope. The astaxanthin-enriched extract was fast analysed in liquid state with the DeltaNu Advantage 532 Raman spectrometer with 532  nm excitation line and 10  s integration time set via NuSpec software.

### X-ray powder diffraction

Shell fragments were finely ground in an agate mortar and their mineral composition was verified by X-ray powder diffraction (XRPD), using a Bruker D8 Advance diffractometer in Bragg-Brentano geometry, with a Cu tube with λ_K_ = 0.15418  nm, Ni filter and a LynxEye detector. Corundum (NIST SRM1976a) is used as an internal standard. The data were collected on a 5–64° 2θ interval at a 0.02° 2θ step, measuring each step for 0.5  seconds. The identification of mineral phases was performed with the Diffrac.Eva 2.1 software (Bruker AXS) using the PDF2 (2012) database.

### Scanning electron microscopy and energy-dispersive x-ray spectroscopy

SEM imaging and EDX measurements were accomplished with a SU8230 Hitachi ultra-high resolution cold-field emission scanning electron microscope. The instrument allows for a combination of topographical and compositional information at a feature resolution of up to 1  nm in optimal conditions. Pieces of claw shells were dried in oven at 40 °C and kept in a container with CaCl_2_ bottom layer to avoid atmospheric water absorption. Before analysis, samples were adherently placed on Hitachi stub SEM holders (aluminium holder with M4 threads covered with carbon discs of 3  mm thickness). A Quorum Q150T sputtering sample coater capable of gold sputtering of controlled thickness for high resolution imaging, and evaporating carbon for EDX analysis was employed. Gold coating thickness was 10  nm (density 19.32  g/m^3^) at a rate of 14  nm/min. An Oxford energy-dispersive x-ray module (Oxford, UK) was used for elemental analysis of shells.

### Statistical analyses

were made using unpaired t-test on GraphPad software and one way analysis of ANOVA (one way ANOVA) between groups. Standard deviation was calculated using Excel software. For statistical relevance, values of p ≤ 0.05 were considered significant, p ≤ 0.01 very significant, p ≤ 0.001 extremely significant.

We first conducted one way ANOVA to four groups (white, blue, green, red) and within certain groups (comparing for certain characters) there were no significant differences. Then we compared series of two groups by ANOVA and by t-test as well, to differentiate the significance between groups. The results were the same (p values) using t test or using ANOVA. Multiple t test was not used here.

## Supplementary information

Supplementary Information.

## Data Availability

The data that support the findings of this study are available within the manuscript, its supplementary information and from the corresponding author upon reasonable request.
