# Supplementary material for: Color-specific porosity in double pigmented natural 3d-nanoarchitectures of blue crab shell
Source: Sci Rep. 2020 Feb 20;10:3019. doi: 10.1038/s41598-020-60031-4 (PMC7033127; doi:10.1038/s41598-020-60031-4)
Supplement: Supplementary file 1 — Supplementary Information. [file 41598_2020_60031_MOESM1_ESM.docx]

**Supplementary Information**

**Color-specific porosity in double pigmented natural 3d-nanoarchitectures of blue crab shell**

Fran Nekvapil^1,2^, Simona Cintă Pinzaru^1,2^*, Lucian Barbu–Tudoran^3,4^, Maria Suciu^3,4^, Branko Glamuzina^5^, Tudor Tamaș^6^, Vasile Chiș^1,2^

^1^Department of Biomolecular Physics, Babeş-Bolyai University, Kogălniceanu 1, 400084 Cluj-Napoca, România

^2^Institute for Research, Development and Innovation in Applied Natural Science, *Fantanele 30, 400327 Cluj-Napoca, Romania*

^3^Electron Microscopy Centre, Babeș-Bolyai University, Clinicilor 5-7, 400006 Cluj-Napoca, România

^4^INCDTIM, Donat 67-103. PO 5 Box 700 400293 Cluj-Napoca, România

^5^Department of Aquaculture, University of Dubrovnik, Ćira Carića 4, 20 000 Dubrovnik, Croatia

^6^Department of Geology, Babeş-Bolyai University, Kogălniceanu 1, 400084 Cluj-Napoca, România

*Corresponding author email: [simona.cinta@phys.ubbcluj.ro](mailto:simona.cinta@phys.ubbcluj.ro)

**
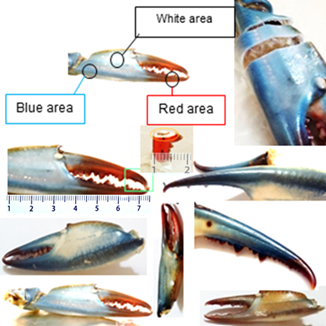
 A)
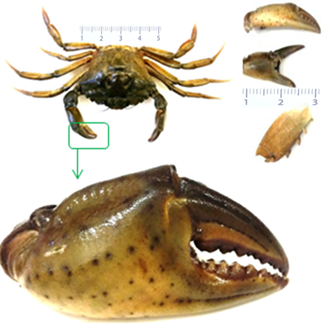
 B)**

**
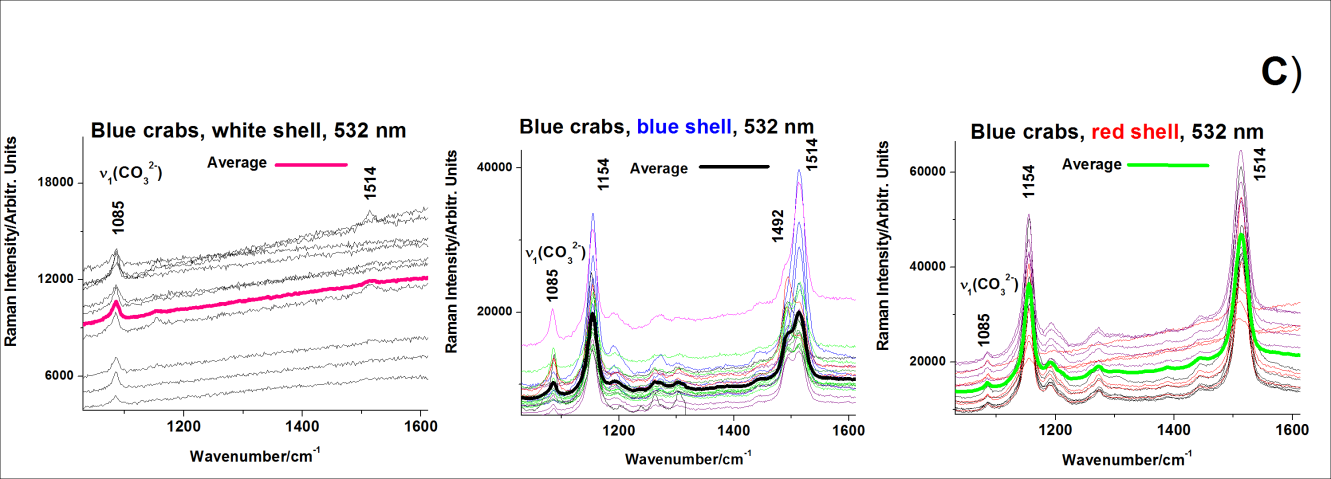
**

**Supplementary Fig. 1.** Photographs of several studied crab shells showing the diversity of color hues and their patterns. Morphological regions from which samples were taken are indicated as blue, red and white areas on *Callinectes sapidus* (the Atlantic blue crab) claws (A), and green area on *Carcinus aestuarii* (the Mediterranean green crab) claws (B). Inserted scales unit: 1 cm. Raman signal taken from white, blue or red shell fragments collected from 10 individuals (5 males, 5 females -2 red claws) using the same laser excitation line (532 nm) and 1 s acquisition, 1 accumulation (C). The average signal is highlighted in each case. The same bands position is observed among individuals. The relative intensity variability of the pigments band versus carbonate stretching mode (1085 cm^-1^) is rather dependent on the local roughness of the shell surface for the incident laser spot and the local interplay of the two pigments ATX and ncb-ATX under focus. Natural accumulation of pigments as a function of crab age (all mature crabs) was not aimed here.

**Supplementary Table S1.** The type, number of samples and experimental purpose of all the samples used in the present study.

|  |  |  | Number of measurements per specimen | | | |
| --- | --- | --- | --- | --- | --- | --- |
| Crab species^1^ | Number of specimens used | Shell^2^ color types | Micro-Raman spectroscopy  (532, 632.8, 785 nm) | FT-Raman | XRD | SEM-EDX |
| *Callinectes sapidus* the Atlantic blue crab | 5 males | blue, white | > 20 on each shell color type and each wavelength | 1  1 | 1  1 | 1  1 |
|  | 5 females | red, blue, white | > 20 on each shell color type and each wavelength | 1  1  1 | 1  1  1 | 1  1  1 |
|  |  | 2 dark red | 10^a^ | 1 | 1 | 1 |
| *Carcinus aestuarii*  the Mediterranean green crab | 3 (not sexed) | green | > 20 | 1 | 1 | 1 |
| Number of measurements = specimens x number of measurements per specimen | | | > 1700 | 28 | 28 | 28 |

^1^Crab individuals were collected on several occasions throughout 2017 and 2018; ^2^A shell contained multiple individual fragments of same color type from the same animal;

^a^Measurements of “dark red shell color type” were consistent with measurements of “red shell color type” in terms of Raman bands presence and positions, and thus can be considered as analogous for micro-Raman spectroscopy analysis.

**
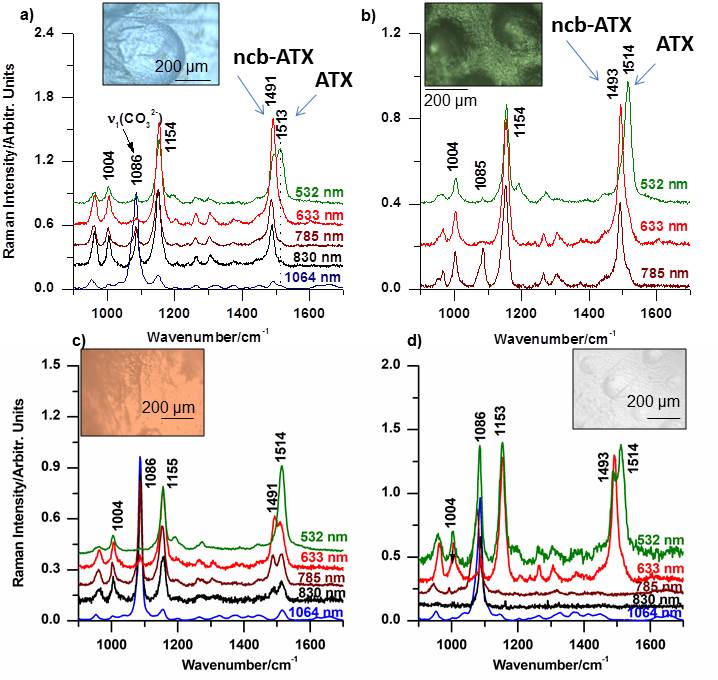
**

**Supplementary Fig. 2.** Multi-laser micro-Raman spectra acquired from a) blue, b) green, c) red, d) white claw shells, showing the co-existence of the free- and non-covalently bonded ATX in all the shell color types (random, poor and noise in white), respectively. Main carotenoid bands along with the CO_3_^2-^ mode (carbonate stretching) are labeled. Spectra were background subtracted, normalized to the carbonate band (1086 cm^-1^) and stacked for clarity. Corresponding micrographs (insets) taken from the shell surface under 200x magnification highlight the intricate morphology and inhomogeneous color intensity. Laser excitation line is given on each spectrum. Exposure: 10 s, 1 accumulation, 20 mW. For FT-Raman (1064 nm) 300 scans were co-added; laser power: 350 mW.


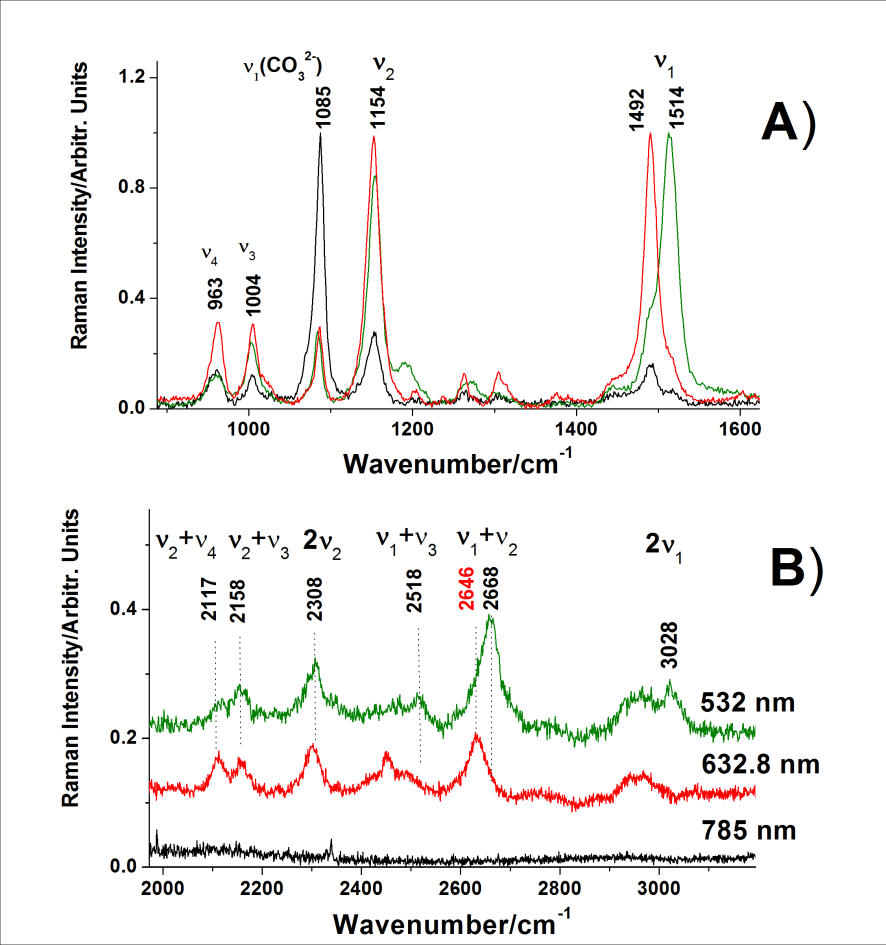


**Supplementary Fig. 3.** Blue cuticle resonance Raman spectra of the free ATX excited with 532 nm (green spectrum) or ncb-ATX excited with 632.8 nm (red spectrum) in the fingerprint range (A) and the corresponding overtones and linear combinations in the 2000-3200 cm^-1^ range (B). Note the absence of these spectral features in the non-resonantly excited cuticle signal with 785 nm from b). Color codes are similar in A) and B). Exposure: 10 s, 1 accumulation, 20 mW.

**
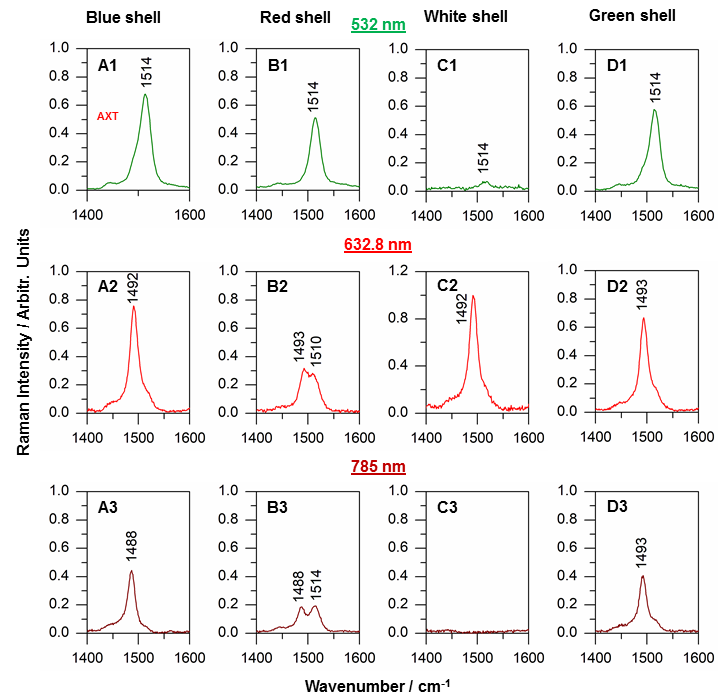
**

**Supplementary Fig. 4.** Comparative display of the carotenoid *v*_1_ Raman band positions in spectra acquired from the four shell types (vertically) with the three laser lines (horizontally), as indicated. Spectra are background-subtracted and normalized to the strongest signal. It can be clearly observed that all shell types contain both free- and non-covalently bonded astaxanthin (ncb-ATX), according to the bands at 1514 cm^-1^ revealed by 532 nm laser line in each shell (top row), and around 1492-1493 cm^-1^ revealed by 632.8 nm line (middle row). The 785 nm laser line is non-resonant to either ATX or ncb-ATX, thus, it excites the normal Raman scattering of both coexistent pigments (bottom panels).

**

**

**Supplementary Fig. 5.** Theoretical DFT calculated Raman spectra of isolated (gas-phase) astaxanthin (red line), and non-covalently bound astaxanthin in aqueous solution (blue line).

**
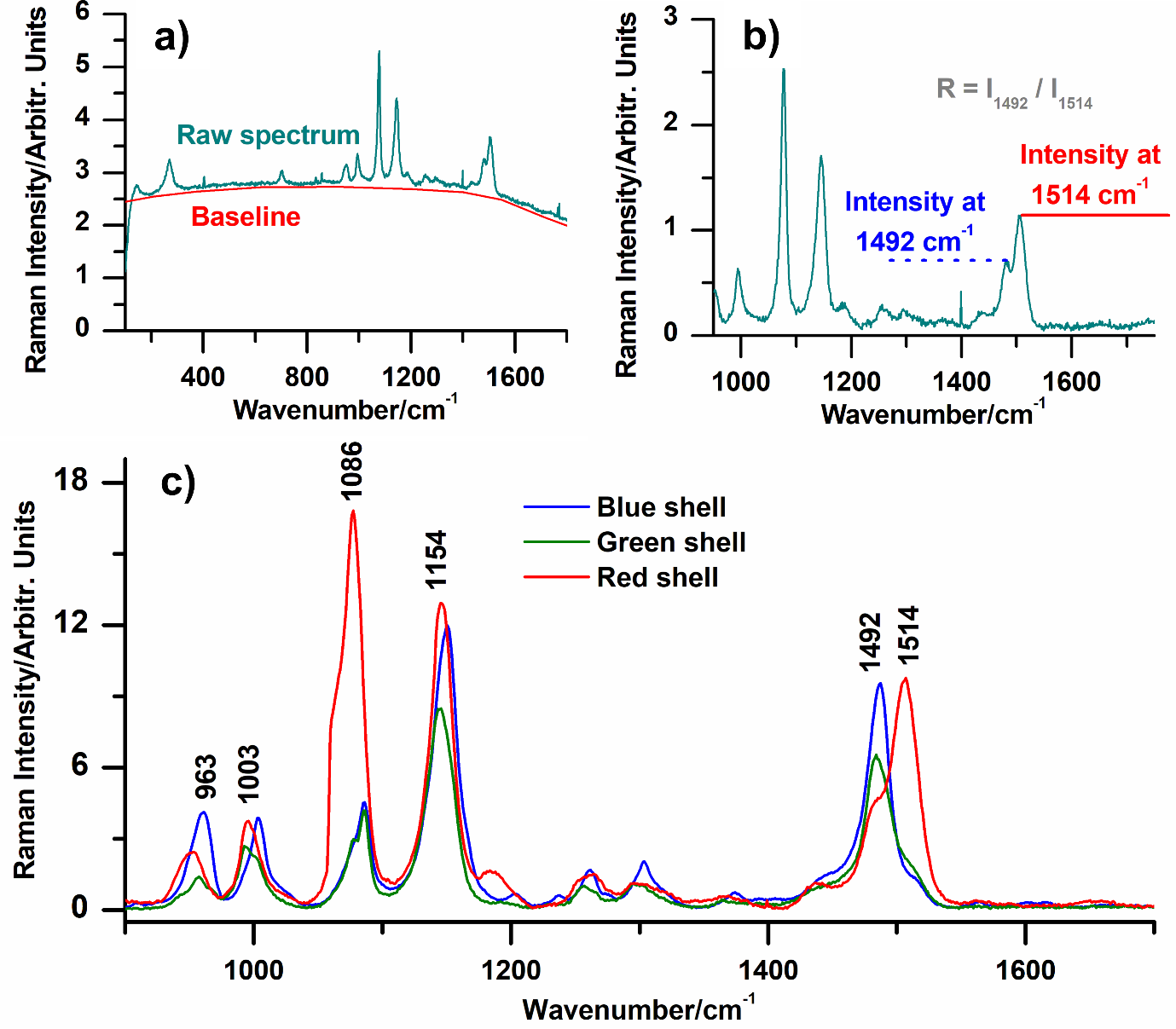
**

**Supplementary Fig. 6.** Procedure of calculating pigment mode ratios using Raman spectra acquired from blue, red and green claw shells non-resonantly excited with the 785 nm line. a) baseline is removed from spectra, b) intensity of ncb-ATX mode around 1492 cm^-1^ and astaxanthin mode at 1514 cm^-1^ is measured. The ratio is calculated as R = I_1492_ / I_1514_. c) average, baseline subtracted spectra normalized to CO_3_^2-^ mode around 1086 cm^-1^, from the three shell color types, as indicated. Red spectrum has been scaled up (x4) for better visibility of the v1(C=C) mode of ATX and ncb-ATX. Note that the crustacyanin contribution to *v*_1_ is the highest in blue shells, exhibiting the highest R value, followed by green shells, while the red cuticle exhibits stronger contribution from free ATX. Exposure: 10 s, 1 accumulation, 20 mW.

**
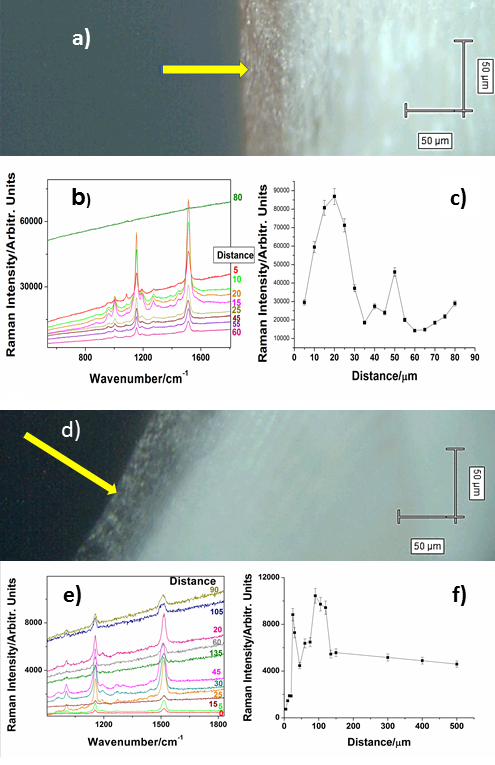
**

**Supplementary Fig. 7.** Depth profiles of the ATX *v*_1_ band intensity in the cross section of (a-c) *Callinectes sapidus* red claw shell, and (d-e) *Carcinus aestuarii* green claw shell. Micrographs (a, d) taken via Raman microscope during measurements show the cross-section planes of respective cuticles, featuring pale endocuticle, below the intensively colored exocuticle. Raman spectra (b, e) were taken along the transect line, from outer layer toward interior (arrow direction), as long as any notable signal-to noise was observed, using the 532 nm excitation line and 100 x objective. Exposure: 10 s, 1 accumulation, 20 mW. Carotenoid content, proportional with the intensity of the *v*_1_ Raman band (c, f), displays a variable distribution with a maximum at 10 to 20 μm below the cuticle surface in both shell types, followed by another peak at the depth of about 50 μm in red and 100 μm in green shell. The data suggest lower, but consistent carotenoid presence towards endocuticle inner layer. Error bars show the standard deviation.

**
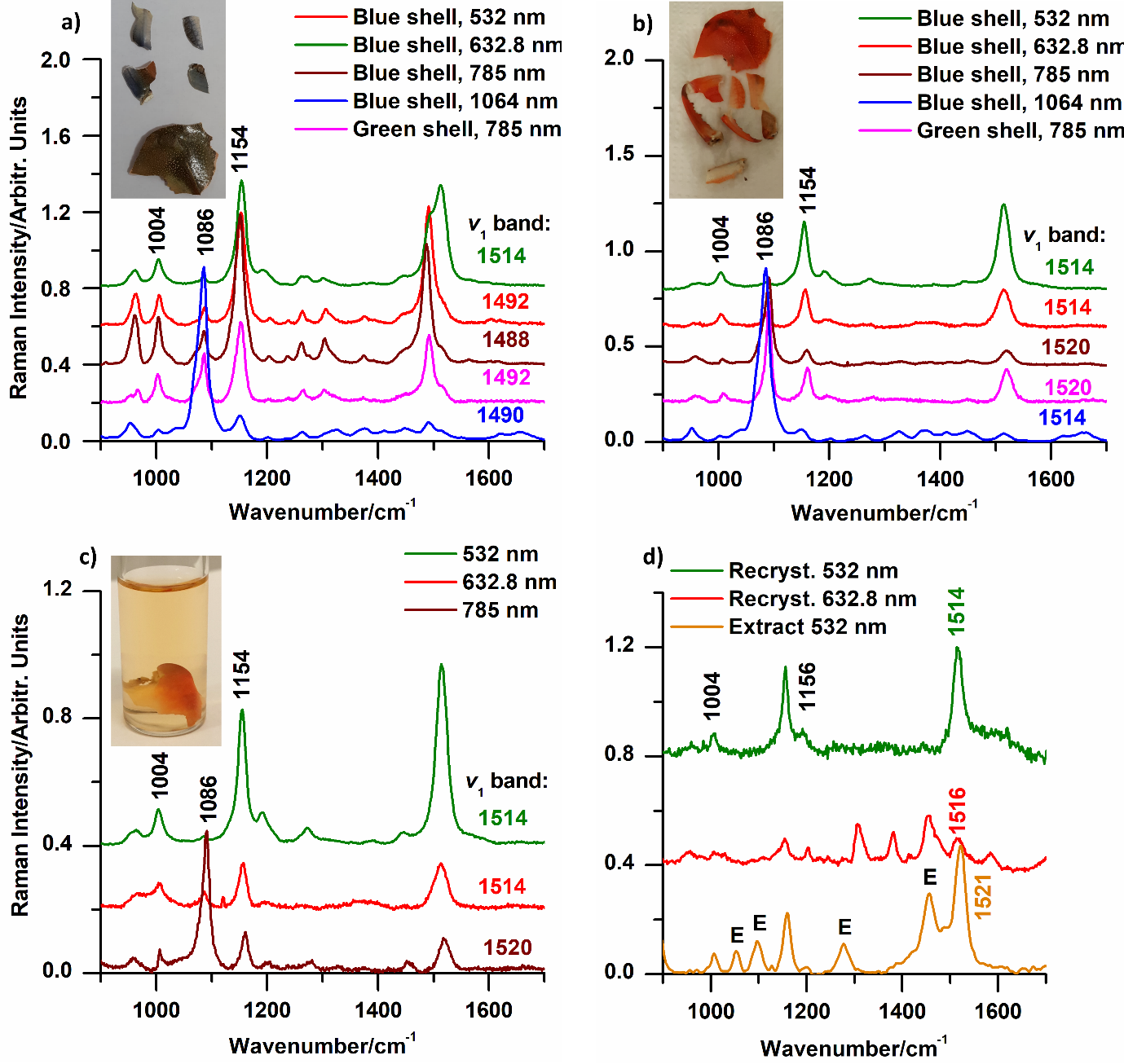
**

**Supplementary Fig. 8.** Raman spectra **(b**ackground-subtracted, averaged) of *C. Sapidus* blue cuticle a) in native state and b) after boiling in hot water (100 °C) for 30 s. Note the disappearance of the ncb-ATX Raman mode at 1492 cm^-1^ in Raman spectra collected from boiled relative to native shells, indicating complete denaturation of AXT-crustacyanin complexes followed by change of shell color from blue to orange (respective insets); c) native blue shell after 14 days of extraction in ethanol; note the absence of the mode assigned to ncb-ATX in crustacyanin and pink-yellowish color of astaxanthin-enriched ethanol extract in the inserted photo; d) shows that ethanol solvent was enriched with carotenoids, which could be again recrystallized after ethanol evaporation (E - ethanol bands). Excitation: 1s, 1 accumulation, 100 mW (solids) and 10 s, 1 accumulation, 200 mW (liquids).

**

**

**Supplementary Fig. 9. A)** FT-Raman spectra collected from 1-blue, 2-red, 3-white and 4-green crab cuticle comparatively shown with the standard calcite and chitin spectra. B) Detail of the 1400-1780 cm^-1^ range with the C=C mode in ATX at 1514 cm^-1^ and ncb-ATX at 1492 cm^-1^ in each coloured cuticle, along with the carbonate 2ν_2_ mode at 1746 cm^-1^. Note the disappearance of the 1492 cm^-1^ band in the blue cuticle after immersion in boiling water. C) Details of the main carbonate mode (including its corresponding FWHM data for each cuticle color code); D) Lorentz fit of the broaden carbonate mode in blue shell, showing the crystalline calcite at 1085 and amorphous phase at 1075 cm^-1^. Their area ratio was r= area_(1085)_/area _(1075)_=2.82. Excitation: 1064 nm, 300 scans, 350 mW.


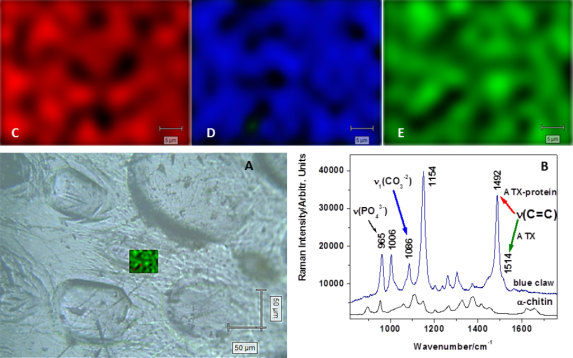


**Supplementary Fig. 10.** Mapping the signal-to-baseline distribution in blue cuticle (A) over an area of 50 μm x 40 μm exploiting its NIR-Raman spectrum excited with 785 nm (B) for carotenoproteins (band at 1492 cm^-1^, C), calcite (1086 cm^-1^, D) and free ATX (1514 cm^-1^, E). The used bands are marked with red, blue or green arrays in B). Note the different pattern distribution of the respective bands intensity, over the mapped area, suggesting high inhomogeneity of the chemical composition at micrometer scale and the interplay of the mineral and organic phase, consistent with the morphology revealed by SEM. Scale bar: 5 μm (C, D, E).


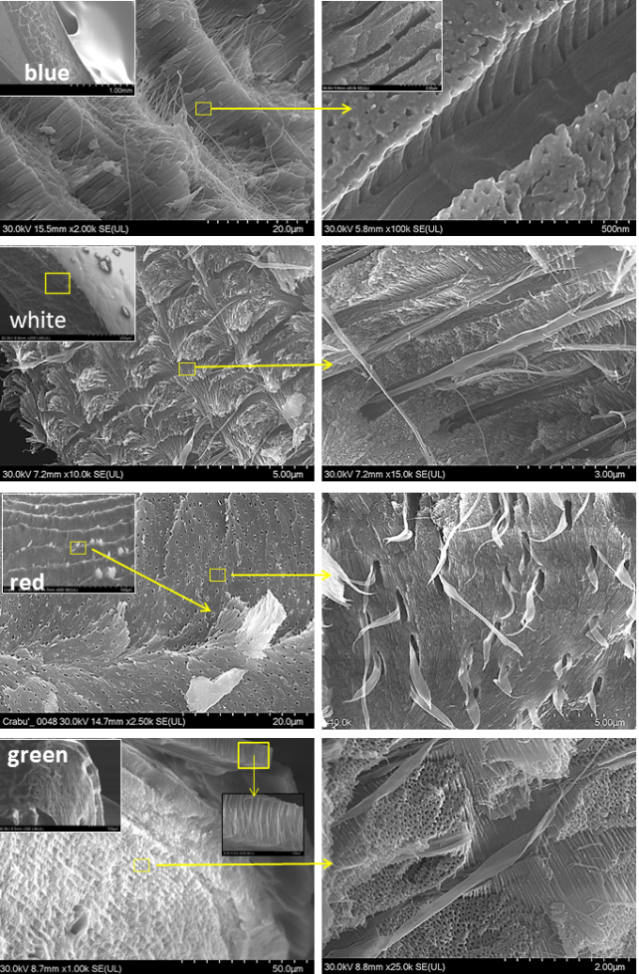
 **
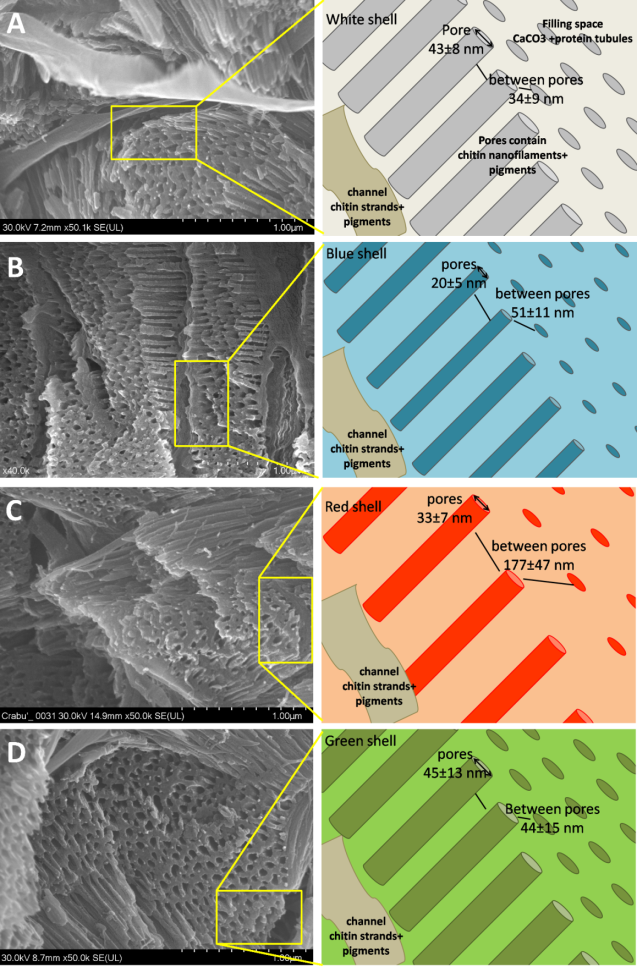
**

**Supplementary Fig. 11. Left:** Representative SEM images showing the morphology of the blue, white, red cuticle of *Callinectes sapidus* and green *Carcinus aestuarii* cuticle, as indicated. The yellow squares locate the higher magnification details highlighting the chitin-protein bundles, their corresponding canals and the regular arrays of nanopillars separated by pores in the ultrastructured canal walls, particularly illustrated in blue, white and green cuticle. Top view of the canals and the helical fibrils arising from them is highlighted in “red” cuticle. **Right:** Additional morphological details of crab cuticle nanoarchitecture from *C. sapidus* white (A), blue (B) and red (C), and *Carcinus aestuarii* green (D) claw shells. SEM images showing grating arrangement of pores and canals are shown along with their schematic representation, to highlight the pores diameter and inter-distances as super-grating, specific for each colour.





**Supplementary Fig.12.** Comparative display of the averaged thickness of the cuticle layers, showing the thinnest epicuticle in blue shell. To calculate averaged data, at least 30 measurements have been conducted in distinct SEM images of layers. Error bars show the standard deviation.


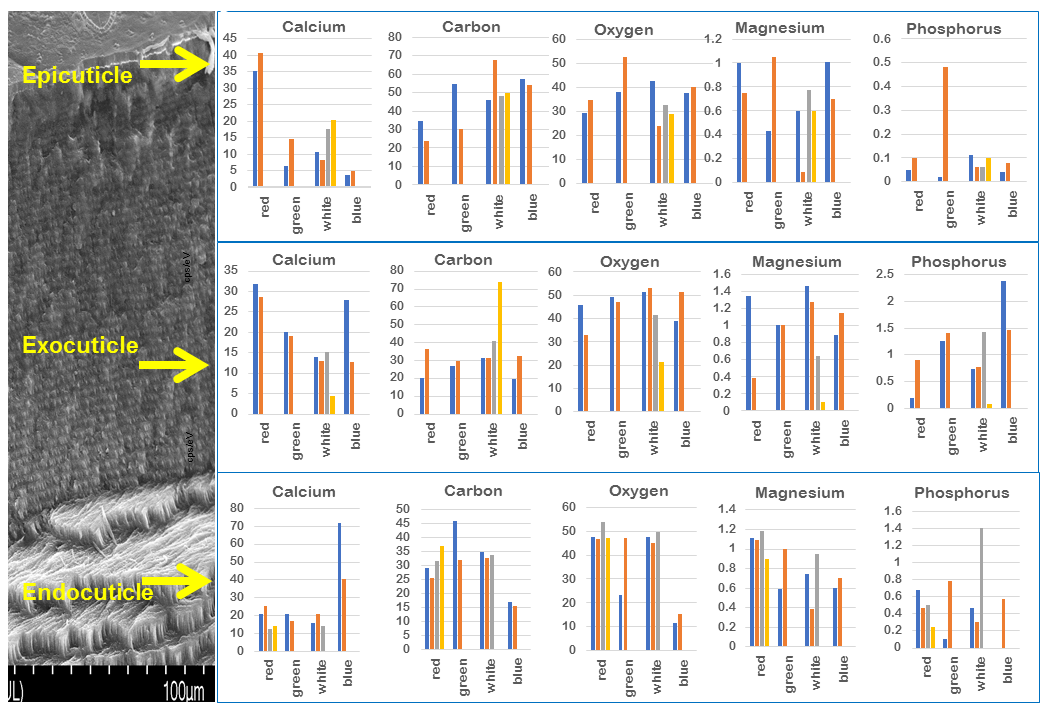


**Supplementary Fig. 13.** Relative mass fractions (wt%) of the main five elements (Ca, C, O, Mg and P, as indicated) in crabs claw shells. EDX data were obtained from 2 to 4 scans corresponding to individual column color in epicuticle, exocuticle and endocuticle of *Callinectes sapidus blue, red and white* and *Carcinus aestuarii* green cuticle color, respectively.

**Supplementary Table S2.** Relative weight contribution (wt%) of all elements recorded in blue, red and white *C. sapidus* and green *C. aestuarii* claw cuticles extracted from the EDX measurements.

| **Cuticle color, layer** | **Green** | | | **Red** | | | **Blue** | | | **White** | | |
| --- | --- | --- | --- | --- | --- | --- | --- | --- | --- | --- | --- | --- |
| **Element** | **endo** | **exo** | **epi** | **endo** | **exo** | **epi** | **endo** | **exo** | **epi** | **endo** | **exo** | **epi** |
| **C** | 38.705 | 28.26 | 42.45 | 30.555 | 28.38 | 28.965 | 15.64 | 26.005 | 55.75 | 33.646 | 44.29 | 52.97 |
| **O** | 40.04 | 48.315 | 45.21 | 48.885 | 39.26 | 31.96 | 26.89 | 45.135 | 38.78 | 47.443 | 41.915 | 31.947 |
| **Na** | 0.52 | 0.815 | 0.585 | 0.577 | 0.35 | 0.21 | 0.22 | 0.315 | 0.205 | 0.37 | 0.405 | 0.217 |
| **Mg** | 0.795 | 1.01 | 0.74 | 1.07 | 0.86 | 0.875 | 0.665 | 1.02 | 0.855 | 0.693 | 0.87 | 0.512 |
| **P** | 0.45 | 1.33 | 0.25 | 0.485 | 0.54 | 0.075 | 0.285 | 1.92 | 0.06 | 0.74 | 0.7475 | 0.082 |
| **K** | 0.2 | 0.21 | 0.065 | 0.062 | 0.17 | 0.065 | 0.27 | 0.24 | 0.035 | 0.14 | 0.0866 | 0.06 |
| **Ca** | 18.955 | 19.605 | 10.6 | 18.342 | 30.29 | 37.86 | 56.03 | 25.365 | 4.32 | 17.06 | 11.667 | 8.18 |
| **S** |  |  |  |  |  |  |  |  |  |  | 0.16 |  |
| **Cl** | 0.34 | 0.46 | 0.1 | 0.07 | 0.29 |  |  |  |  |  |  |  |

*Abbreviations*: endo – endocuticle, exo – exocuticle, epi - epicuticle.
